# Polymorphisms at Locus 4p14 of Toll-Like Receptors *TLR-1* and *TLR-10* Confer Susceptibility to Gastric Carcinoma in *Helicobacter pylori* Infection

**DOI:** 10.1371/journal.pone.0141865

**Published:** 2015-11-11

**Authors:** M. Ravishankar Ram, Khean Lee Goh, Alex Hwong Ruey Leow, Bee Hoon Poh, Mun Fai Loke, Richard Harrison, Esaki M. Shankar, Jamuna Vadivelu

**Affiliations:** 1 Department of Medical Microbiology, Faculty of Medicine, University of Malaya, Lembah Pantai, 50603, Kuala Lumpur, Malaysia; 2 Department of Medicine, Faculty of Medicine, University of Malaya, Lembah Pantai, 50603, Kuala Lumpur, Malaysia; 3 Department of Pathology, BP Diagnostics Center Sdn. Bhd., 30250, Ipoh, Perak, Malaysia; 4 Life Science Research Division, Bio-Rad Laboratories Pty. Ltd., Gladesville, New South Wales, 2111, Australia; 5 Tropical Infectious Diseases Research and Education Center (TIDREC), Department of Medical Microbiology, Faculty of Medicine, University of Malaya, Lembah Pantai, 50603, Kuala Lumpur, Malaysia; University of the Pacific, UNITED STATES

## Abstract

*Helicobacter pylori* (*H*. *pylori*) *-*induced gastric inflammation impacts the functions of leptin- and ghrelin-producing cells in the gastroduodenum. Inflammation resulting from *H*. *pylori* sensing via Toll-like receptors (TLRs) and the associated downstream signaling largely remain ambiguous. Here, we investigated the role of gut hormones, pro-inflammatory cytokines and single nucleotide polymorphisms (SNPs) associated with TLR 4p14 in *H*. *pylori* disease in 30 subjects with non-ulcer dyspepsia (NUD), 40 with peptic ulcer disease (PUD) and 15 with gastric cancer (GC) subjects positive and negative for *H*. *pylori* infection. The level of pro-inflammatory cytokines was directly proportional to the severity of gastritis, and disease status influenced the levels of gut hormones and pro-inflammatory cytokines. *TLR-1* SNPs *rs4833095 and TLR-10* SNPs *rs10004195* and were directly associated with *H*. *pylori* disease, and were up-regulated in the presence of *H*. *pylori* in a genotype-independent manner. We concluded that *TLR-1 rs4833095* and *TLR10 rs10004195* confer susceptibility to development of gastroduodenal disease, especially GC in *H*.*pylori* disease.

## Introduction

*Helicobacter pylori* (*H*. *pylori*) is the major infectious cause of chronic gastritis, and plays a key role in the development of gastric cancer (GC) [1]. Inflammation of the gastric compartment associated with *H*. *pylori* infection reportedly afflicts numerous gastric cell types, especially cells that secrete gastric hormones, leptin and ghrelin [2]. Previous studies have suggested that gastric colonization of *H*. *pylori* could be associated with differential circulating levels of leptin [3–5], and anti-*H*. *pylori* therapy has been shown to improve gastric absorption functions [6–8]. Evidence also suggests that *H*. *pylori* could regulate the levels of leptin and ghrelin [9, 10]. Moreover, the pathogenicity of *H*.*pylori* appears to be largely dependent on genetic heterogeneity [11]. It is also becoming increasingly clear that several specific host genes are involved in response to *H*. *pylori* colonization, immune escape and gastric mucosal injury.

Epithelial cells of the gastric mucosa are amongst the first cellular barriers for *H*. *pylori* in the gastrointestinal tract [12], which mainly recognize *H*. *pylori-derived* microbe-associated molecular patterns (MAMPs) through ligation of pattern recognition receptors (PRRs), especially the toll-like receptors (TLRs) [13, 14]. The PRRs recognize bacterial lipopolysaccharides (LPS) to effect the secretion of proinflammatory responses [15]. Hence, the early stages appear to be the most important phase in the establishment of an infection when the pathogen is most susceptible to destruction by the host innate immune system. Hence, the early phase offers the best opportunity for therapeutic interventions against microbial pathogens. *H*. *pylori* displays a plethora of MAMPs, which needs due recognition by PRRs of the host to mount an immune response. Recent investigations have shown that TLR-1-TLR-6-TLR-10 locus is associated with increased levels of anti-*H*. *pylori* antibodies generated via TLR-1 [15]. Further, others suggest that TLR-10 also could play an important role in *H*. *pylori* infection. The association between polymorphisms associated with TLRs, especially TLR-2 and severity of intestinal metaplasia in *H*. *pylori* disease has also been reported [16], and their potential role as a risk-factor for development of GC [17]. Here, we hypothesized that certain polymorphisms involving *TLR-1* and *TLR-10* associated with the recognition of *H*. *pylori* likely contribute to progression of GC and increased susceptibility to *H*. *pylori* infection and GC in some individuals than others [18]. We also selected the TLR locus on 4P14, and the lead single nucleotide polymorphisms (SNPs) rs10004195 and rs4833095 to study the association between ghrelin and leptin, as well as circulating pro-inflammatory cytokine levels with the genetic factors implicated with susceptibility to gastroduodenal diseases and GC following *H*. *pylori* infection.

## Materials and Methods

### Human Subjects

Total study subjects of 95 patients, tissue samples were available from 85 patients, and tissue samples were not available from five asymptomatic *H*.*pylori* positive and five asymptomatic *H*.*pylori* negative subjects. In ELISA studies involving 85 subjects who underwent gastroscopy at the endoscopy unit of the University of Malaya Medical Centre (UMMC). Of these, 15 were diagnosed and classified as *H*. *pylori*-positive non-ulcer dyspepsia (NUD), 15 as *H*. *pylori*-negative NUD, 34 as *H*. *pylori*-positive peptic ulcer disease (PUD), six as *H*. *pylori*-negative PUD, eight as *H*. *pylori*-positive gastric cancer (GC), seven as *H*. *pylori*-negative GC, five as *H*. *pylori*-positive without gastroduodenal symptoms, and five as *H*. *pylori*-negative with no gastroduodenal disease. Fifty five were females and 40 were males. We further subdivided the study subjects into subgroups A (18–40 years old) and B (41–80 years old) (Table 1). In TLR SNPs studies involving 85 subjects which is the same as the ELISA study disease status samples and the asymptomatic samples were not included. Of these, 15 were diagnosed and classified as *H*. *pylori*-positive non-ulcer dyspepsia (NUD), 15 as *H*. *pylori*-negative NUD, 34 as *H*. *pylori*-positive peptic ulcer disease (PUD), six as *H*. *pylori*-negative PUD, eight as *H*. *pylori*-positive gastric cancer (GC), and seven as *H*. *pylori*-negative GC. Body mass index (BMI) was defined as weight in kilograms divided by the square of height in meters. BMI ≥28.0 for men and ≥28.8 for women was considered as obese as per the published criteria of the World Health Organization (WHO) [19]. All the methods were carried out in compliance with the approved guidelines, and all experimental protocols were approved by the Medical Ethics Committee (MEC) of University of Malaya Medical Centre (UMMC), Malaysia. The current work was strictly conducted in accordance with the International Conference on Harmonization Guidelines and 1962 Declaration of Helsinki. Written informed consents were obtained from all patients and healthy controls or their legal representatives as a voluntary participation before enrolment into the investigations.

**Table 1:** Demographic and laboratory characteristics of the study population. Numbers in parentheses indicate percentage.

Characteristics	Asymptomatic(*H*.*pylori* infection)(N = 10)		Non-ulcer dyspepsia (NUD)(*H*.*pylori* infection)(N = 30)		Peptic ulcer disease (PUD)(*H*.*pylori* infection) (N = 40)		Gastric cancer (GC)(*H*.*pylori* infection) (N = 15)	
*H*. *pylori* status	Positive	Negative	Positive	Negative	Positive	Negative	Positive	Negative
Numbers	5 (50)	5 (50)	15 (50)	15 (50)	34 (85)	6 (15)	8 (53.3)	7 (46.6)
Female	3 (30)	3 (30)	8 (26.6)	8 (26.6)	18 (45)	3 (7.5)	3 (20)	4 (26.6)
Male	2 (20)	2 (20)	7 (23.3)	7 (23.3)	16 (40)	3 (7.5)	3 (20)	4 (26.6)

### Plasma

Blood samples were collected in BD Vacutainer^®^ tubes (BD, Franklin Lakes, NJ, USA) from each subject after overnight fasting, and were centrifuged within 30 mins of collection. Plasma samples were aliquoted and frozen at -80°C for subsequent measurement of circulating gut hormones/hormone receptors and pro-inflammatory cytokines.

### *H*. *pylori* Culture

Tissue biopsies from both gastric antrum and corpus were extracted during routine gastroscopy for rapid urease test (RUT), culture for *H*. *pylori* and histopathological investigations. Gastric biopsies for *H*. *pylori* culture were homogenized in different tubes and plated directly on non-selective and selective chocolate agar supplemented with 7% lysed horse blood (Oxoid, UK), and contained vancomycin (10μg/ml), amphotericin B (5μg/ml), trimethoprim (5μg/mL) and nalidixic acid (20μg/mL) (Sigma, USA). The inoculated plates were incubated for 3–10 days in a humidified 10% CO_2_ incubator at 37°C.

### Histopathology

We conducted histological examination of 85 biopsy specimens categorized under three different disease statuses, viz., NUD, PUD, GC (*H*. *pylori* positive, n = 62; *H*. *pylori* negative, n = 33) classified based on infiltration of neutrophils, mononuclear cells, atrophy and progression to intestinal metaplasia and graded on a four-point scale basis: 0, absent; 1, mild; 2, moderate; and 3, severe (S1 Data)—histological examination of 85 biopsy specimens. Hematoxylin and eosin (H & E) stained tissue biopsy samples were reviewed by an independent certified histopathologist (BHP) who was blinded to clinical diagnosis and *H*. *pylori* infection status of the patients.

### ELISA

Serological investigations (ELISA) were performed on 85 subjects that underwent gastroscopy at the endoscopy unit of UMMC. In total, ELISA was performed on 95 subjects. Serum leptin, leptin receptor, ghrelin, ghrelin receptor, insulin, soluble insulin receptor (sIR), TNF-α, TNFR-2, IFN-γ, neuropeptide Y, IL-4, IL-6, IL-8 and IL-10 concentrations were measured as per the manufacturer’s instructions using commercial double-antibody sandwich ELISA Kits (Diagnostic Systems Laboratories, Webster, TX, USA).

### Single Nucleotide Polymorphisms

Electronic databases (PUBMED, Scopus, Science Direct, Ovid, Biosis Previews, Scirus databases, CINAHL, IMBIOMED, Scielo and LILACS) were used to search for polymorphisms involved in the TLR signaling pathway that were associated with cancer, infectious disease or were functionally relevant.

### Genotyping

Each individual included in the study, genomic DNA was extracted from peripheral whole blood samples using the QIAamp Blood DNA Mini Kit as described by the manufacturer (Qiagen; Hilden, Germany). DNA was rehydrated in sterile water and normalized to 10 ng/μL for customized SNP genotyping of *TLR-1* SNPs *rs4833095 and TLR-10* SNPs *rs10004195* in TLRs (Assay I.D: C_44103606_10, RS Number: rs4833095 and Assay I.D: C_29979325_10, RS Number: rs10004195). TaqMan^®^ SNP Genotypig Assays (Applied Bio systems, Waltham, MA, USA) through the application of ddPCR was carried out using the QX100 Droplet Digital PCR system (Bio-Rad Laboratories, Hercules, CA, USA). ddPCR reactions were 20 μL aqueous volumes that contained final concentrations of 1x ddPCR Super mix (Bio-Rad), 1x TaqMan probes, 1 U/μL Hind III and 10 ng/μL of genomic DNA. Droplet generation and droplet reading for ddPCR were carried out according to the manufacturer’s instructions using Bio-Rad reagents. The thermal cycling profile was enzyme activation at 95°C for 5 min followed by 40 cycles of (denaturation at 94°C for 30 sec and annealing/ extension at 58°C for 60 sec; 2°C/sec ramp rate), final extension at 98°C for 10 min and held at 4°C 1°C/sec ramp rate. Data analysis was carried out using Quanta Soft software version 1.3.2.0 (Bio-Rad).

### Statistical Analysis

The odds ratios (OR) and 95% confidence intervals (CI) were calculated by means of the Fisher’s exact probability test (two-tailed p-values). Multivariate statistical analyses were performed using a binary logistic regression (LR) adjusted by *H*. *pylori* status and gender. P-values < 0.05 indicated statistical significance. Bonferroni correction was used for multiple testing of the TLR alleles. The data was analyzed by means of the programs SPSS version (SPSS Inc., Chicago, USA).

## Results

### Subjects Positive for *H*. *pylori* with Underlying Gastric Cancer Showed Increased Levels of Plasma Leptin and Decreased Levels of Soluble Insulin Receptor

We found that the levels of plasma leptin were significantly increased in all the three disease groups as compared to the asymptomatic cases by a commercial double-antibody sandwich ELISA (Diagnostic Systems Labs, Webster, TX, USA). No significant association was observed between the symptomatic age-subgroups A and B, and the asymptomatic control age-subgroups A and B in regards to leptin receptor, ghrelin, ghrelin receptor, insulin, sIR and neuropeptide-Y and pro-inflammatory cytokines. Interestingly, sIRs were significantly down-regulated as compared to the asymptomatic controls (Table 2). Next, we analyzed the three different disease statuses (NUD, PUD, GC) positive and negative for *H*. *pylori* infection between the different subgroups A and B. However, for all the three disease statuses in *H*. *pylori* positive subjects, leptin, leptin receptor and pro-inflammatory cytokines, we did not observe any significant difference relative to *H*. *pylori* negative subjects.

**Table 2:** Distribution (mean and standard deviation) of gut hormones and its cognate receptors and pro-inflammatory cytokine concentrations based on age subgroups.

ELISA	Age subgroup A	Age subgroup B	One-way ANOVA(p value)
Leptin (ng/ml)	6.4 ± 3.2	8.5 ± 2.1	0.036†
Leptin receptor (ng/ml)	111.5 ± 45.4	127.4 ± 31.1	0.100
Ghrelin (pg/ml)	472.5 ± 64	446 ± 36.6	0.130
Ghrelin receptor (ng/ml)	59.1 ± 12.3	50.6 ± 5.4	0.156
Insulin (mU/l)	29.8 ± 10.7	21.1 ± 2.9	0.240
Insulin receptor (ng/ml)	69.7 ± 15.3	56.1 ± 3.4	0.050†
Neuropeptide Y (ng/l)	360.5 ± 25.4	320.7 ± 14.4	0.510
IFN-γ (ng/l)	120.1 ± 54.3	162.8 ± 8.5	0.767
TNF-α (ng/l)	554.2 ± 50.7	715.9 ± 60.9	0.721
TNFR-2 (pg/ml)	221.2 ± 123.6	265.8 ± 103.8	0.462
IL-4 (pg/ml)	142.5 ± 87.4	176.6 ± 71	0.415
IL-6 (ng/l)	311.4 ± 150.5	386.2 ± 116.4	0.673
IL-8 (ng/l)	72.5 ± 58.7	83.5 ± 57.9	0.477
IL-10 (pg/ml)	280.3 ± 169.5	329.7 ± 140.6	0.293

†Significant correlation

### Gastric Cancer and Peptic Ulcer Disease Patients with *H*. *pylori* Infection Displayed Increased Levels of Pro-Inflammatory Cytokines

**Fig 1:**
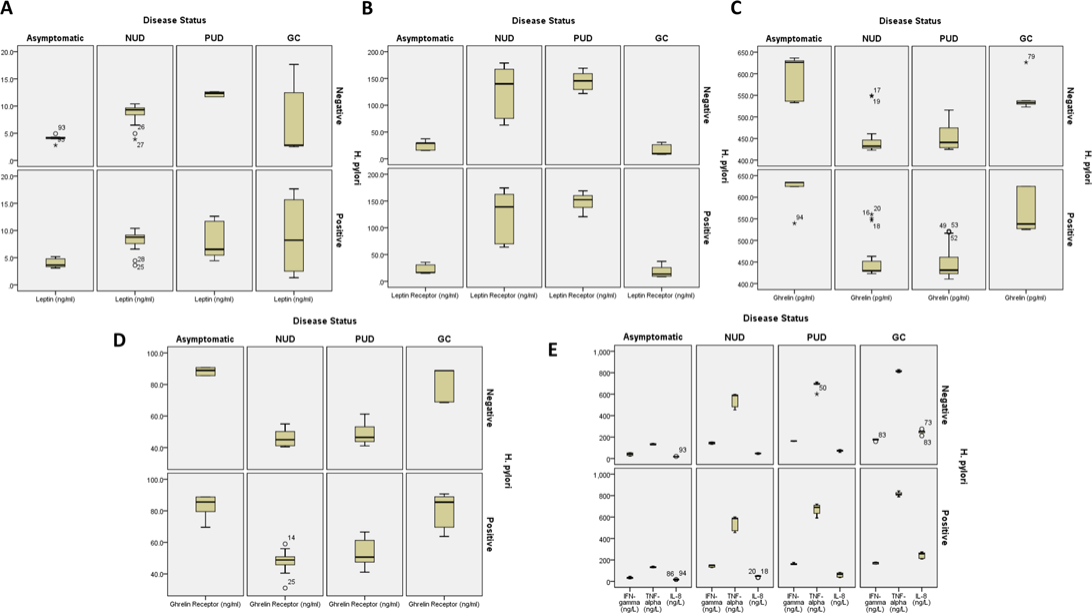
Whiskers plot analysis of distribution of plasma immunoreactive gut hormones (A) Leptin (B) Leptin receptor (C) Ghrelin (D) Ghrelin receptor (E) pro-inflammatory cytokine concentrations (TNF-α, IFN-γ, IL-8) in gastroduodenal diseases and gastric cancer (GC). Plasma leptin, leptin receptor, ghrelin, ghrelin receptor, TNF-α, IFN-γ and IL-8 concentrations measured using commercial double-antibody sandwich ELISA. Data were from three independent experiments. Level of significance set at p<0.05. (Note: IL, interleukin; IFN-γ, interferon gamma; TNF-α, tumor necrosis factor alpha)

Our clinical interpretation analysis showed that for all three disease statuses, the levels of leptin and leptin receptor were significantly increased in PUD and GC as compared to NUD and asymptomatic cases but that were positive for *H*. *pylori* infection. Similarly, the levels of all pro-inflammatory cytokines were significantly elevated in the PUD and GC subjects as compared to NUD and asymptomatic *H*. *pylori* positive cases. However, the plasma levels of ghrelin, ghrelin receptor, insulin, sIR and neuropeptide Y were significantly down-regulated in GC and gastroduodenal disease subjects (Fig 1A–1E and Table 3).

**Table 3:** Distribution (mean and standard deviation) of gut hormones and its respective receptors and pro-inflammatory cytokine concentrations based on disease status (asymptomatic, NUD, PUD, GC).

ELISA	Asymptomatic	Non-ulcer dyspepsia (NUD)	Peptic ulcer disease (PUD)	Gastric cancer (GC)	One-way ANOVA (p value)†	Post hoc Multiple correlation
Leptin (ng/ml)	2.27 ± 0.71	5.1 ± 2.1	6.2 ± 2.8	8.5 ± 3.3	0.000	0.831**
Leptin receptor (ng/ml)	23.5 ± 9.7	109.8 ± 38	131.5 ± 30.1	135.8 ± 19.2	0.000	0.879**
Ghrelin (pg/ml)	580.4 ± 50.7	477.2 ± 58.8	454.6 ± 35.4	435.1 ± 24.9	0.000	-0.732**
Ghrelin receptor (ng/ml)	69.9 ± 8.1	54.2 ± 6.9	53.9 ± 7.6	57 ± 8.3	0.000	-0.613**
Insulin (mU/l)	38.7 ± 7.8	27.5 ± 8.7	24.7 ± 7.5	29.4 ± 9	0.000	-0.672**
Insulin receptor (ng/ml)	83.4 ± 9.9	62.3 ± 7	57.5 ± 5	58.4 ± 5.3	0.000	-0.841**
Neuropeptide Y (ng/l)	377.9 ± 13.6	331.2 ± 10.8	319.4 ± 13.7	328.0 ± 13.9	0.000	-0.941**
IFN-γ (ng/l)	42.2 ± 11.5	149.7 ± 9.8	162.5 ± 8	168.6 ± 8.4	0.000	0.876**
TNF-α (ng/l)	133.5 ± 9.3	557.6 ± 62.2	687.7 ± 37.2	799 ± 23.3	0.000	0.944**
TNFR-2 (pg/ml)	58.5 ± 15.5	168.7 ± 27.2	277.8 ± 31.1	518.7 ± 32.3	0.000	0.945**
IL-4 (pg/ml)	33.4 ± 16.6	129.6 ± 42.9	174.4 ± 54.3	286.2 ± 13.6	0.000	0.907**
IL-6 (ng/l)	78.7 ± 16.7	250.3 ± 75.9	422.8 ± 55.3	507.9 ± 41	0.000	0.961**
IL-8 (ng/l)	15.3 ± 5.7	52.3 ± 11.3	80.8 ± 32.3	238.5 ± 23.4	0.000	0.796**
IL-10 (pg/ml)	33 ± 7.3	199.3 ± 46.2	337.9 ± 62.9	584.1 ± 64.4	0.000	0.945**

**Correlation is significant at the 0.05 level (2-tailed)

†All statistically significant

### Plasma Leptin and Pro-Inflammatory Cytokines were Markedly Enhanced in Symptomatic *H*. *pylori* Infection, and Correlated Positively with Gut Hormone Levels

Next, we measured the plasma levels of leptin, ghrelin and insulin, sIR, neuropeptide Y and all the pro-inflammatory cytokines in patients of three different disease statuses (Table 3). We found that the levels of leptin and all the pro-inflammatory cytokines were significantly increased in symptomatic *H*. *pylori* positive cases (NUD, PUD and GC) as compared to asymptomatic, but positive for *H*. *pylori* infection (*p*<0.001). Interestingly, the cytokine levels were also significantly higher in symptomatic *H*. *pylori* negative cases relative to asymptomatic *H*. *pylori* negative cases (*p*<0.001). Of note, leptin, leptin receptor, and all the pro-inflammatory cytokines were the highest among *H*. *pylori* positive GC cases (*p*<0.001).

Multiple inter-correlation analyses showed that the levels of leptin and leptin receptor showed strong positive inter-correlation between pro-inflammatory cytokines (Fig 1A–1E) in all the disease statuses investigated. Similarly, ghrelin and ghrelin receptor also showed strong positive correlation with insulin, sIR and neuropeptide Y in these groups. Leptin and leptin receptor correlated inversely with insulin, sIR and neuropeptide Y. Likewise, ghrelin and ghrelin receptor correlated inversely with all the pro-inflammatory cytokines studied. Together, we found that plasma leptin and pro-inflammatory cytokine levels were markedly enhanced in symptomatic *H*. *pylori* infection, and correlated positively with leptin and ghrelin levels.

### Ghrelin Concentration Correlated Negatively and Leptin Positively with Inflammation of the Antral and Corpus Compartment

Next, we conducted histological examination of 85 biopsy specimens categorized under three different disease statuses, viz., NUD, PUD, GC (*H*. *pylori* positive, n = 62; *H*. *pylori* negative, n = 33) classified based on infiltration of neutrophils, mononuclear cells, atrophy and progression to intestinal metaplasia and graded on a four-point scale basis: 0, absent; 1, mild; 2, moderate; and 3, severe (data not shown). For all three disease statuses, serum leptin, leptin receptor, pro-inflammatory cytokine levels were significantly higher in mild, moderate and severe NUD, PUD, GC cases positive for *H*. *pylori* infection compared to those negative for *H*. *pylori* infection (*p*<0.001). On the other hand, serum ghrelin, ghrelin receptor, insulin, sIR and neuropeptide Y levels were significantly lesser in mild, moderate and severe NUD, PUD, GC cases positive for *H*. *pylori* infection compared to *H*. *pylori* negative subjects (*p*<0.001).

The antral and corpus gastritis scores were significantly higher in *H*. *pylori*-positive relative to *H*. *pylori*-negative cases for NUD (mean, 3.3±1.7 vs. 0.8±1.3; *p*<0.001), PUD (2.7±1.5 vs. 0.5±0.9; *p*<0.001) and GC 2.1±1.2 vs. 0.3±0.4; *p*<0.0001) respectively. In particular, inflammation and activity scores were higher both for *H*. *pylori*-positive NUD, PUD and GC cases involving either the antrum (*p*<0.001 and *p*<0.001) or the corpus (*p*<0.0001 and *p*<0.05). The mean serum ghrelin levels differed significantly according to the severity of gastritis. As such, grades of inflammation involving the antrum (237±4.16; *p*<0.01 and *p*<0.05) as well as the corpus (205±4.02; *p*<0.01 and *p*<0.05 respectively) were negatively associated with ghrelin concentrations within both *H*. *pylori*-positive and -negative patient groups compared to leptin antral (6.71±2; *p*<0.01 and *p*<0.05) as well as corpus inflammation (8.67±1.4; *p*<0.01 and *p*<0.05 respectively) scores were positively associated with leptin concentrations within both *H*. *pylori*-positive and -negative groups (S1 Data).

### Degree of Inflammation Correlated Positively with Neutrophil Infiltration at the Corpus and Antrum, and was Higher in Subjects with Underlying *H*. *pylori* Infection

**Fig 2:**
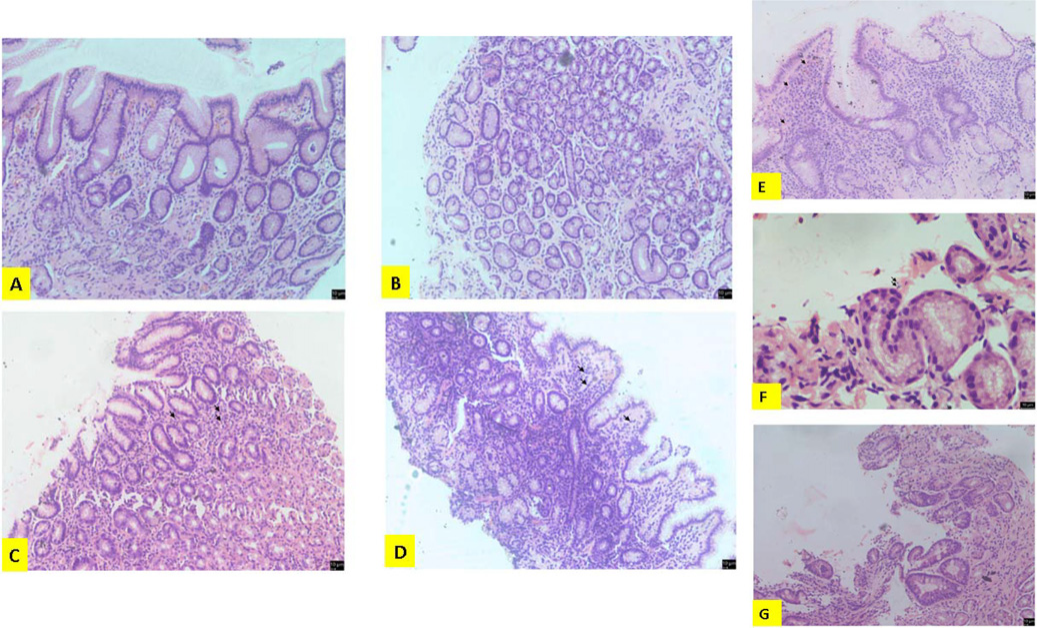
Hematoxylin & Eosin (H & E) histopathological investigations on gastric antrum and corpus mucosal tissues of individuals with different gastroduodenal diseases and gastric cancer (GC) positive and negative for *H*. *pylori* infection. (A & B) Antrum tissue showing chronic changes with mild glandular atrophy (Magnification: 100X). (**C**) Corpus tissue showing signs of chronic active gastritis with mild chronic inflammatory activity and moderate active inflammation. There is moderate infiltration of neutrophils (arrow) (Magnification: 100X). (**D**) Antrum tissue showing chronic active gastritis with marked chronic inflammatory activity. There is marked infiltration of plasma cells and lymphocytes (arrow) (Magnification: 100X). (**E**) Antrum tissues showing chronic active gastritis with marked chronic inflammatory activity and superimposed moderate active inflammation. There is moderate glandular atrophy and intestinal metaplasia. There is infiltration of eosinophils (arrow) (Magnification: 100X). (**F**) Antrum tissue showing marked intestinal metaplasia with goblet cell change (Magnification: 100X). (**G**) Gastric tissue with *H*. *pylori* (arrow) (Magnification: 400X).

Next, we investigated the cellular mediators of inflammation at the corpus and antral regions of the tissue specimens of different patient groups. We found that the degree of inflammation correlated positively with infiltration of neutrophils and mononuclear cells at the corpus and antrum regions, and was higher in NUD and PUD subjects with underlying *H*. *pylori* infection (Fig 2A–2G). Further, glandular atrophy and intestinal metaplasia at the corpus was significantly increased in cancer subjects (grade 2.28, *p*<0.005 and grade 6.14, *p*<0.001, respectively), although only intestinal metaplasia showed a significant increase, with only the antrum specimens (grade 4.15, *p*<0.001).

### Disease Status-Wise Relationship Between TLR Polymorphisms and *H*. *pylori* Infection

Next, we compared between the asymptomatic, GD and GC groups for demographic data that included disease status and *H*. *pylori* infection wise **(** Table 1 **)**. We found that *H*. *pylori* infection was significantly associated with GD and GC than asymptomatic controls (*p*<0.005). The heterogeneity measures for the TLR SNPs were 71.3% for *rs10004195* and 65% for *rs4833095* (Table 4). Notably, there was a significant association between SNPs *rs10004195* and *rs4833095* (*p*<0.0028).

**Table 4:** Allele analysis of TLR-1 (*rs4833095*) and TLR-10 (*rs10004195*) in the study population.

Gene	Polymorphism	Nucleotide change	Allele analysis						Odds ratio (OR)	95% Confidence interval (95% CI)	p value
			Non-ulcer dyspepsia (NUD)		Peptic ulcer disease (PUD)		Gastric cancer (GC)				
			X	Y	X	Y	X	Y			
***TLR-1***	*rs4833095*	T>C	21	9	21	19	8	7	1.28	0.43–2.88	0.0042
***TLR-10***	*rs10004195*	T>A	21	9	21	19	8	7	0.86	0.34–1.65	0.0056

### TLR-1 SNP *rs10759932* C Allele Significantly Increased the Risk of *H*. *pylori* Infection

Next, we sought to investigate the allele and genotype frequencies for TLR SNPs *rs10004195* and *rs4833095* (Tables 4 & 5). We found that individuals with r*s10004195* T allele were not protected against GC development (*p*<0.0036). However, we found that the *rs4833095* C allele was a risk factor for the onset of GC. Together, we found that TLR SNPs *rs10004195* AT and *rs4833095* CT alleles were not protective against GC.

**Table 5:** Genotype analysis of single nucleotide polymorphisms in the study population.

Polymorphism	*H*. *pylori* status	Genotype	Disease status			Odds ratio (OR)	95% Confidence interval (95% CI)	p value
			Non-ulcer dyspepsia (NUD)	Peptic ulcer disease (PUD)	Gastric cancer (GC			
***TLR rs4833095***	Negative	CC	5	2	2	0.72	0.42 – 1.14	0.0042
	Negative	CT	5	2	1	0.83	0.46–1.63	0.0057
	Negative	TT	5	2	4	0.73	0.34–1.32	0.0059
	Positive	CC	7	14	2	1.65	0.90–3.56	0.0034
	Positive	CT	5	10	4	1.46	0.65–3.42	0.0038
	Positive	TT	3	10	2	1.23	0.72–2.48	0.0058
***TLR rs10004195***	Negative	AA	3	3	3	0.62	0.20–1.30	0.0048
	Negative	AT	7	2	2	0.69	0.32–1.36	0.0036
	Negative	TT	5	1	2	0.90	0.39–1.84	0.0050
	Positive	AA	9	12	3	1.28	0.46–2.42	0.0032
	Positive	AT	3	12	3	0.95	0.64–2.10	0.0046
	Positive	TT	3	10	2	0.76	0.36–1.45	0.0042

Given that *H*. *pylori* is a known major risk factor for the onset of GC, next we examined the effect of genetic polymorphisms of on *H*. *pylori* infection. We found that TLR-1 *rs10759932* C allele significantly heightened the risk of involvement of *H*. *pylori* in GC (*p*<0.0056; OR, 0.59; 95% CI, 0.41–0.86) as compared to the PUD (*p*<0.0056; OR, 0.59; 95% CI, 0.41–0.86) and NUD (*p*<0.0056; OR, 0.59; 95% CI, 0.41–0.86) (Table 5 **)**.

### TLR SNPs *rs4833095* and *rs10004195* Resulted in Increased Levels of Leptin, IFN-γ and IL-8 in *H*. *pylori* Positive Patients with Gastric Cancer

**Fig 3:**
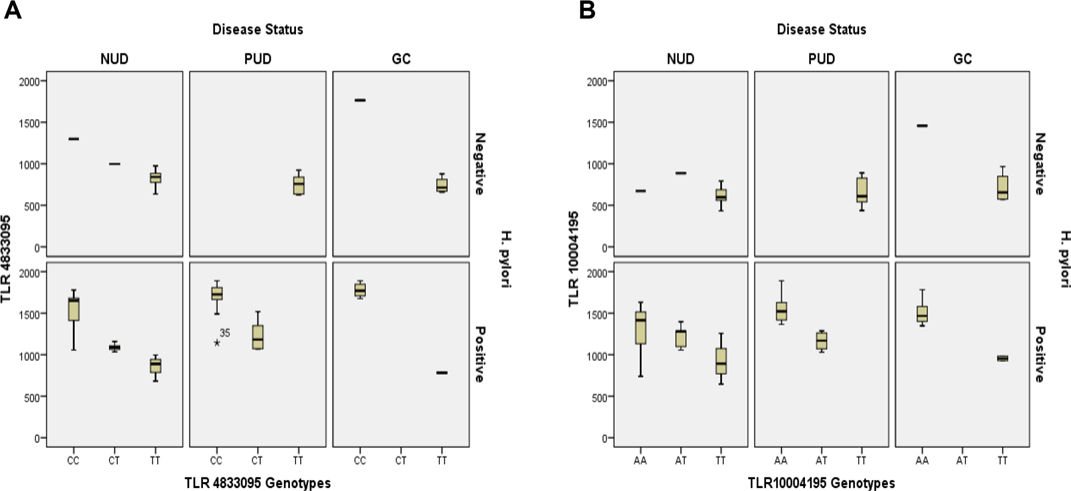
Boxplot analysis of single nucleotide polymorphism (A) TLR-1 *rs4833095* and (B) TLR-10 *rs10004195* in gastroduodenal diseases and gastric cancer (GC). Single nucleotide polymorphism TLR-1 *rs4833095* and TLR-10 *rs10004195* were measured using ddPCR. Data were from three independent experiments. Level of significance set at p<0.05. (Note: ddPCR, digital droplet polymerase chain reaction)

Next, we performed multiple inter-correlation analyses using Pearson’s correlation coefficient between plasma levels of gut hormones leptin and ghrelin, pro-inflammatory cytokines IFN-γ and IL-8 concentration and SNPs *rs10004195* and *rs4833095* in GD and GC cases. Our boxplot analysis have indicated that these polymorphisms contribute to translational regulation of TLRs, (Fig 3A & 3B) haplotype CC and AA of *rs10004195* AA and *rs4833095* CC were increased in GC irrespective of *H*. *pylori* infection. TLR SNPs *rs4833095* and *rs10004195* resulted in increased levels of leptin, IFN-γ and IL-8 in GD and GC cases.

## Discussion

Our investigations have clearly shown a significant correlation between leptin, leptin receptor and pro-inflammatory cytokine levels. We also found an inverse correlation between the levels of ghrelin and severity of gastritis due to *H*. *pylori*. This implies that production of ghrelin likely has an impact on development of GC in the presence of *H*. *pylori* infection [20]. This is concurrent to previous reports that the distribution of ghrelin-secreting cells in the gastric mucosa was significantly lesser in *H*. *pylori*-infected patients compared to healthy controls, whereas in patients *H*. *pylori* infection an inverse correlation was seen between ghrelin and inflammation [21]. Recent experimental evidence suggests that both plasma ghrelin and leptin concentrations were significantly reduced in *H*. *pylori*-positive relative to *H*. *pylori*-negative cases [22].

Classification of age-subgroups with underlying GD and GC showed an increase in the levels of plasma leptin and decrease of sIR. Others have shown that the levels of plasma leptin were significantly elevated in individuals with existing GDs [23]. Here we observed no significant association between the symptomatic age-subgroups A and B in regards to leptin receptor, ghrelin, ghrelin receptor insulin, sIR and neuropeptide Y and pro-inflammatory cytokines as compared with the asymptomatic control age-subgroups [24]. Interestingly, significant down-regulation of sIR was evident in the GC cases as compared to asymptomatic controls. Others have shown that elevated plasma leptin could increase the levels of TNF-α in the systemic circulation [25]. One mechanism whereby TNF-α triggers the onset of insulin deficiency is by increasing adipocyte-associated lipolysis that also results in increase of plasma leptin [26], which likely down-regulates ghrelin directly as seen with all the three different disease statuses positive for *H*. *pylori* infection, which also revealed increase of plasma IFN-γ and IL-8 levels. Ghrelin, the later having established anti-inflammatory and anti-apoptotic properties [27] are believed to play a paramount role in the pathogenesis of gastroduodenal diseases and GC. *H*. *pylori* positivity is reportedly associated with differential regulations of leptin and ghrelin, which likely delineates a scenario where defects could contribute to sustenance of *H*. *pylori*-driven pathogenic response [28]. Due to the close relationship between *H*. *pylori* and development of gastritis, it is conceivable that infection could lead to the onset of atrophic gastritis and intestinal metaplasia. There have been instances of gastric atrophy with intestinal metaplasia present in association with GC, NUD and PUD in line with our current findings [29]. Generally, these lesions occur decades after acquisition of infection. Potential mechanisms underlying reduced synthesis of ghrelin in the presence of the above pathology could induce functional impairment in ghrelin-secreting cells, possibly mediated by cytokines [30].

Consistent with current findings, recent observations have demonstrated low plasma ghrelin levels in GC with an evolving autoimmune gastric pathology [31]. This is of interest due to the effects *H*. *pylori* infection has on gastric or circulating ghrelin dynamics in gastric and duodenal ulcer. In the present study, we observed an inverse correlation between ghrelin and leptin, although the influence of these factors on the levels of each other remains to be investigated. Our current study has shown that in GC, PUD and NUD-associated with *H*. *pylori*-induced gastritis, the levels of ghrelin and leptin responses appears to be dependent on one another to show significant differences in the prevalence of TLR on 4P14, the lead SNP was rs10004195, closely followed by rs4833095 across gastroduodenal and GC, a finding likely to explain the significant association between rs10004195 and rs4833095 in *H*. *pylori* infection. In disease status analysis GC, significant associations were identified between rs10004195 and rs4833095 in *H*.*pylori* infected subjects.

Our results have clearly shown that the inhibitory effect of ghrelin on pro-inflammatory cytokines, which also supports the likely regulatory role of ghrelin receptor, leptin and leptin receptor in controlling cytokine-induced gastroduodenal disease. Moreover, the degree of inflammation correlated positively with neutrophil and mononuclear cell infiltration at the corpus and antrum, and was higher in subjects with underlying *H*. *pylori* infection with the concerted role of IL-8 and leptin has also been shown to inhibit ghrelin levels in *H*. *pylori* positive cases. Our data on the direct effect of leptin on GC, neutrophils and mononuclear cells indicate a beneficial effect of leptin, which induces the production of the IL-8, mainly by monocytes, to such an extent that it could increase IFN-γ production. This could be important for GC because IFN-γ has been shown to be detrimental for this disease [32], whereas IL-8 could be beneficial [33]. In this context, increase of leptin to potentially induce IL-4 or IL-6 production by patient- or control-derived cells is unexpected. Another study has shown that leptin could induce the secretion of IL-8 by human monocytes [34] to exert anti-inflammatory effects in clinical GC [35]. It is therefore possible that leptin, like IL-6, and IL-10, has bystander immunomodulatory functions [36]. This is indicative of the existence of a reciprocal regulatory network through which ghrelin and leptin regulates inflammation. Moreover, ghrelin also exerts potent anti-inflammatory effects and attenuates in gastroduodenal diseases, supporting the potential use of agonists of both the gastric hormones in the management of gastric disorders. Our results showed significant inverse association between the levels of plasma ghrelin and development of GC. Furthermore, we also found that individuals naïve for GC but with extensive atrophic gastritis had significantly lesser levels of plasma ghrelin. The results of the current study provide evidence for plasma ghrelin being a surrogate marker of gastric mucosal function in GC in the study groups in our current setting. The PRR-induced pro-inflammatory signaling can initiate innate immune responses, which are required for the activation of adaptive immune responses [37, 38]. To initiate these responses, the transcription factor TLR locus on 4P14, the lead SNP was rs10004195, closely followed by rs4833095 play pivotal roles due to their abilities to facilitate the release of pro-inflammatory mediators, namely IL-8 and IFN-γ. However, a controlled mechanism of regulation is paramount for strong transient responses, which is achieved via amplification, as well as down-regulation during the later stages of infection [39–41]. It is notable that regulation of gene expression at the transcriptional level plays an important role in the post-transcriptional mechanisms regulating the decay of mRNAs encoding various gut hormones leptin, leptin receptor and pro-inflammatory molecules IFN-γ, TNF-α, IL-8 also contribute to achieve this goal. First, the TLR on 4P14, the lead SNP was *rs10004195*, closely followed by *rs4833095* polymorphism has been associated with gastroduodenal disease and GC with susceptibility to *H*. *pylori*. At the mechanistic level, this may be explained by the mutation causing reduced ghrelin, IL-10 levels in response to *H*. *pylori*, thereby resulting in diminished IFN-γ-mediated TNF-α response. The second functional TLR variant is associated with an increased risk of developing GC, in agreement with TLRs recognizing PAMPs from these two pathogens. Given the association of SNPs rs4833095 and rs10004195 with leptin levels in *H*.*pylori*-induced PUD and also GC, it would be interesting to investigate the role of these SNPs in non-*H*.*pylori*-induced PUD (for instance, drug induced PUD) and GC.

**Fig 4:**
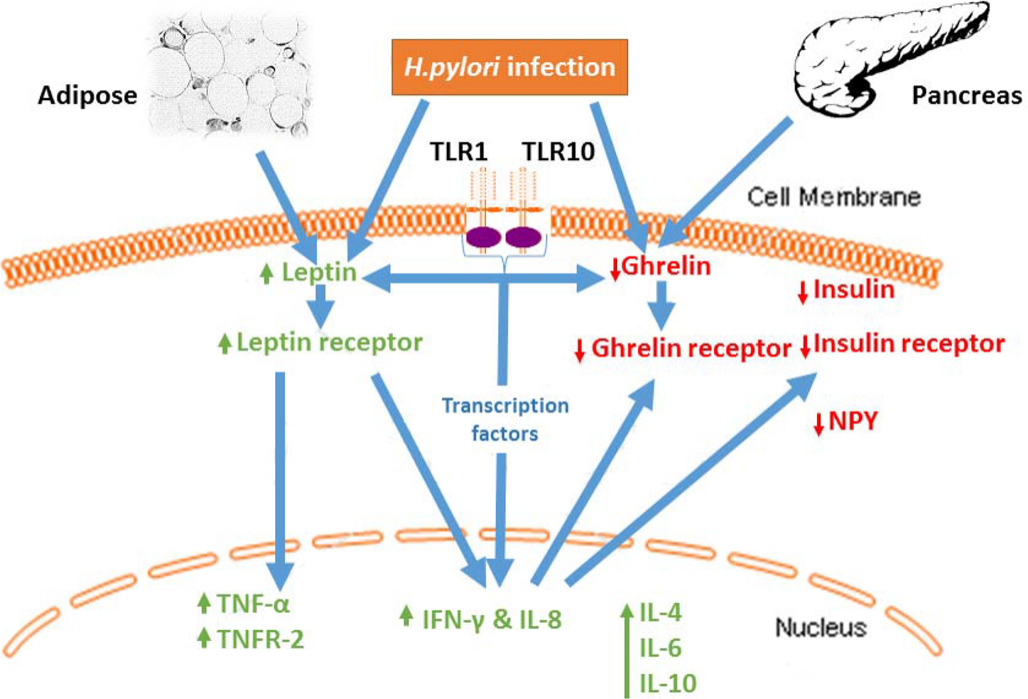
Proposed mechanism of the association of *H*. *pylori* infection with gastroduodenal diseases and gastric cancer (GC). Engagement of surface TLRs with PAMPs leads to activation of transcription factors in the cell nucleus resulting in IFN-γ and IL-8 (CXCL8) by identical pathways. *H*.*pylori* infections have been known to increase leptin levels, which in turn inhibits ghrelin functions by a feedback mechanism. Polymorphisms in TLR-1 and TLR-10 branches of the innate immune system likely modulate the direction and magnitude of host responses. (Note: PAMP, pathogen-associated molecular patterns; TLR, Toll-like receptor; IL, interleukin; IFN-γ, interferon gamma)

In conclusion, our current study clearly supports the role of leptin and ghrelin as markers of gastric mucosal alterations in GC suggestive of increased risk for the onset of GC in *H*. *pylori* infection. Our results provide further insights into the pathogenesis of GC, and may provoke more detailed investigations leading to identification of a panel of diagnostic markers applicable to surveillance programmes. Our findings show the evidence to support the NUD, PUD and GC, and consistent with our hypothesis that, because *H*. *pylori* is initially sensed by TLR signaling, polymorphisms in TLR components could clearly modulate the magnitude of the resultant immune responses. Based on our current findings, we have proposed a mechanism on the association of *H*. *pylori* infection with gastroduodenal diseases and gastric cancer (Fig 4). Engagement of surface TLRs with PAMPs leads to activation of transcription factors in the cell nucleus resulting in IFN-γ and IL-8 by identical pathways. *H*. *pylori* infections have been known to increase leptin levels, which in turn inhibits ghrelin functions by a feedback mechanism. Polymorphisms in TLR-1 and TLR-10 could modulate the magnitude of host immune responses. Together, we concluded that *TLR-1 rs4833095* and *TLR-10 rs10004195* confer susceptibility to development of gastroduodenal disease, especially GC in *H*.*pylori* disease.

## Supporting Information

S1 Data (DOCX)

## Abbreviations

BMI: body mass index
CNS: central nervous system
CI: confidence interval
ddPCR: digital droplet polymerase chain reaction
EDTA: ethylene diamine tetraacetic acid
ELISA: enzyme-linked immunosorbent assay
GC: gastric cancer
GD: gastroduodenal diseases
GH,: growth hormone
GWAS: genome-wide association study
IFN-γ: interferon gamma
IL: interleukin
IR: insulin resistance
LPS: lipopolysaccharide
LR: logistic regression
NF- κB: nuclear factor-kappa B
NUD: non-ulcer dyspepsia
NPY: neuropeptide Y
Ob: obese
OR: odds ratios
PUD: peptic ulcer disease
POMC: pro-opiomelanocortin
TNF-α: tumor necrosis factor alpha
RUT: rapid urease test
SNP: single nucleotide polymorphism
TLR: Toll-like receptor
TNFR-2: tumor necrosis receptor factor– 2
UMMC: University of Malaya Medical Centre
WHR: waist-hip ratio
