# Supplementary material for: Polymorphisms at Locus 4p14 of Toll-Like Receptors TLR-1 and TLR-10 Confer Susceptibility to Gastric Carcinoma in Helicobacter pylori Infection
Source: PLoS One. 2015 Nov 11;10(11):e0141865. doi: 10.1371/journal.pone.0141865 (PMC4641589; doi:10.1371/journal.pone.0141865)
Supplement: S1 Data — (DOCX) [file pone.0141865.s001.docx]

**S1 Data. Correlation study between gut hormones and its respective receptors, pro-inflammatory cytokine concentrations and histological severity in non-ulcer dyspepsia, peptic ulcer and gastric cancer subjects.**

| Gut hormone | *H. pylori* status | **Tissue isolation site** | **Non-ulcer dyspepsia** **(NUD)** | | | | | | | | | | | |
| --- | --- | --- | --- | --- | --- | --- | --- | --- | --- | --- | --- | --- | --- | --- |
|  |  |  | Neutrophils | | | Mononuclear cells | | | Atrophy | | | Intestinal metaplasia | | |
|  |  |  | Mild | Moderate | Severe | Mild | Moderate | Severe | Mild | Moderate | Severe | Mild | Moderate | Severe |
| Mean  Plasma Ghrelin  pg/ml | *H. pylori* positive | Antrum | **423**  **(400-450)** | **356**  **(300-375)** | **237**  **(200-250)** | **437**  **(400-450)** | **374**  **(350-400)** | **216**  **(200-250)** | **417**  **(320-440)** | **344**  **(300-375)** | **249**  **(200-275)** | **462**  **(440-489)** | **384**  **(320-440)** | **238**  **(200-260)** |
|  |  | Corpus | **414**  **(350-450)** | **323**  **(300-375)** | **205**  **(190-240)** | **461**  **(430-490)** | **316**  **(300-340)** | **189**  **(150-200)** | **436**  **(400-450)** | **309**  **(290-340)** | **215**  **(200-235)** | **482**  **(440-500)** | **322**  **(300-340)** | **188**  **(150-200)** |
|  | *H. pylori* negative | Antrum | **426**  **(390-440)** | **381**  **(360-400)** | **258**  **(240-265)** | **414**  **(400-430)** | **396**  **(350-420)** | **227**  **(200-250)** | **399**  **(375-410)** | **374**  **(350-400)** | **242**  **(230-260)** | **412**  **(400-440)** | **377**  **(320-440)** | **245**  **(220-260)** |
|  |  | Corpus | **407**  **(390-420)** | **338**  **(320-350)** | **199**  **(175-220)** | **435**  **(400-450)** | **323**  **(300-350)** | **187**  **(150-210)** | **414**  **(400-430)** | **316**  **(300-340)** | **189**  **(150-210)** | **433**  **(420-450)** | **343**  **(320-360)** | **174**  **(150-210)** |

| Gut hormone | *H. pylori* status | **Tissue isolation site** | **Peptic ulcer disease (PUD)** | | | | | | | | | | | |
| --- | --- | --- | --- | --- | --- | --- | --- | --- | --- | --- | --- | --- | --- | --- |
|  |  |  | Neutrophils | | | Mononuclear cells | | | Atrophy | | | Intestinal metaplasia | | |
|  |  |  | Mild | Moderate | Severe | Mild | Moderate | Severe | Mild | Moderate | Severe | Mild | Moderate | Severe |
| Mean  Plasma Ghrelin  pg/ml | *H.pylori* positive | Antrum | **435**  **(400-450)** | **366**  **(300-380)** | **242**  **(200-274)** | **447**  **(400-474)** | **366**  **(340-380)** | **248**  **(200-275)** | **451**  **(430-490)** | **389**  **(320-440)** | **241**  **(200-275)** | **440**  **(400-450)** | **374**  **(320-440)** | **229**  **(200-250)** |
|  |  | Corpus | **454**  **(430-470)** | **323**  **(300-375)** | **209**  **(190-240)** | **488**  **(450-500)** | **327**  **(300-350)** | **212**  **(200-250)** | **483**  **(450-500)** | **334**  **(320-350)** | **210**  **(190-230)** | **474**  **(450-490)** | **316**  **(300-340)** | **197**  **(175-220)** |
|  | *H.pylori* negative | Antrum | **444**  **(425-460)** | **384**  **(320-440)** | **246**  **(200-274)** | **416**  **(375-450)** | **387**  **(350-420)** | **253**  **(240-265)** | **423**  **(390-440)** | **381**  **(350-400)** | **241**  **(230-260)** | **404**  **(400-440)** | **396**  **(320-440)** | **238**  **(220-260)** |
|  |  | Corpus | **415**  **(390-420)** | **331**  **(320-350)** | **203**  **(175-220)** | **431**  **(400-450)** | **335**  **(300-350)** | **185**  **(150-210)** | **431**  **(400-450)** | **342**  **(300-360)** | **208**  **(150-220)** | **427**  **(420-450)** | **323**  **(300-340)** | **191**  **(150-210)** |

| Gut hormone | *H. pylori* status | **Tissue isolation site** | **Gastric Cancer (GC)** | | | | | | | | | | | |
| --- | --- | --- | --- | --- | --- | --- | --- | --- | --- | --- | --- | --- | --- | --- |
|  |  |  | Neutrophils | | | Mononuclear cells | | | Atrophy | | | Intestinal metaplasia | | |
|  |  |  | Mild | Moderate | Severe | Mild | Moderate | Severe | Mild | Moderate | Severe | Mild | Moderate | Severe |
| Mean  Plasma Ghrelin  pg/ml | *H.pylori* positive | Antrum | **427**  **(400-450)** | **350**  **(300-380)** | **251**  **(200-274)** | **458**  **(400-474)** | **354**  **(340-380)** | **252**  **(200-275)** | **465**  **(430-490)** | **394**  **(320-440)** | **253**  **(200-275)** | **430**  **(400-450)** | **385**  **(320-410)** | **234**  **(200-250)** |
|  |  | Corpus | **444**  **(430-470)** | **338**  **(300-375)** | **227**  **(190-240)** | **476**  **(450-500)** | **335**  **(300-350)** | **228**  **(200-250)** | **472**  **(450-500)** | **339**  **(320-350)** | **220**  **(190-230)** | **463**  **(450-490)** | **323**  **(300-340)** | **182**  **(175-220)** |
|  | *H.pylori* negative | Antrum | **439**  **(425-460)** | **379**  **(320-440)** | **255**  **(200-274)** | **429**  **(375-450)** | **381**  **(350-420)** | **258**  **(240-265)** | **419**  **(390-440)** | **375**  **(350-400)** | **248**  **(230-260)** | **426**  **(400-440)** | **381**  **(320-440)** | **244**  **(220-260)** |
|  |  | Corpus | **410**  **(390-420)** | **325**  **(320-350)** | **210**  **(175-220)** | **442**  **(400-450)** | **337**  **(300-350)** | **190**  **(150-210)** | **427**  **(400-450)** | **356**  **(300-360)** | **189**  **(150-220)** | **434**  **(420-450)** | **331**  **(300-340)** | **187**  **(150-210)** |

| Gut hormone | *H. pylori* status | **Tissue isolation site** | **Nonulcer dyspepsia** **(NUD)** | | | | | | | | | | | |
| --- | --- | --- | --- | --- | --- | --- | --- | --- | --- | --- | --- | --- | --- | --- |
|  |  |  | Neutrophils | | | Mononuclear cells | | | Atrophy | | | Intestinal metaplasia | | |
|  |  |  | Mild | Moderate | Severe | Mild | Moderate | Severe | Mild | Moderate | Severe | Mild | Moderate | Severe |
| Mean Plasma Leptin ng/ml | *H. pylori* positive | Antrum | **4.92**  **(2-7)** | **3.56**  **(2-7)** | **6.71**  **(2-9)** | **4.61**  **(2-9)** | **3.56**  **(2-9)** | **6.71**  **(2-9)** | **4.63**  **(2-9)** | **3.56**  **(2-9)** | **6.71**  **(2-9)** | **4.63**  **(2-9)** | **3.56**  **(2-9)** | **6.71**  **(2-9)** |
|  |  | Corpus | **3.03**  **(2-7)** | **4.26**  **(2-9)** | **8.67**  **(2-9)** | **3.36**  **(2-9)** | **4.26**  **(2-9)** | **8.67**  **(2-9)** | **3.36**  **(2-9)** | **4.26**  **(2-9)** | **8.67**  **(2-9)** | **3.36**  **(2-9)** | **4.26**  **(2-9)** | **8.67**  **(2-9)** |
|  | *H. pylori* negative | Antrum | **2.82**  **(2-7)** | **2.72**  **(2-7)** | **4.43**  **(2-9)** | **2.23**  **(2-7)** | **2.76**  **(2-7)** | **4.43**  **(2-9)** | **2.28**  **(2-7)** | **2.73**  **(2-7)** | **4.43**  **(2-9)** | **2.27**  **(2-7)** | **2.77**  **(2-7)** | **4.43**  **(2-9)** |
|  |  | Corpus | **3.34**  **(2-7)** | **4.12**  **(2-9)** | **6.07**  **(2-9)** | **3.76**  **(2-9)** | **4.12**  **(2-9)** | **6.07**  **(2-9)** | **3.76**  **(2-9)** | **4.12**  **(2-9)** | **6.07**  **(2-9)** | **3.74**  **(2-9)** | **4.12**  **(2-9)** | **6.07**  **(2-9)** |

| Gut hormone | *H. pylori* status | **Tissue isolation site** | **Peptic Ulcer Disease (PUD)** | | | | | | | | | | | |
| --- | --- | --- | --- | --- | --- | --- | --- | --- | --- | --- | --- | --- | --- | --- |
|  |  |  | Neutrophils | | | Mononuclear cells | | | Atrophy | | | Intestinal metaplasia | | |
|  |  |  | Mild | Moderate | Severe | Mild | Moderate | Severe | Mild | Moderate | Severe | Mild | Moderate | Severe |
| Mean Plasma Leptin  ng/ml | *H. pylori* positive | Antrum | **4.61**  **(2-9)** | **3.47**  **(2-9)** | **6.65**  **(2-9)** | **4.54**  **(2-9)** | **3.87**  **(2-9)** | **6.45**  **(2-9)** | **4.65**  **(2-9)** | **3.98**  **(2-9)** | **6.54**  **(2-9)** | **4.32**  **(2-9)** | **3.43**  **(2-9)** | **6.55**  **(2-10)** |
|  |  | Corpus | **3.32**  **(2-9)** | **4.32**  **(2-9)** | **8.88**  **(5-12)** | **3.78**  **(2-9)** | **4.32**  **(2-9)** | **8.98**  **(5-14)** | **3.89**  **(2-9)** | **4.65**  **(2-9)** | **8.54**  **(5-14)** | **3.45**  **(2-9)** | **4.76**  **(2-9)** | **8.76**  **(5-14)** |
|  | *H. pylori* negative | Antrum | **2.29**  **(2-7)** | **2.84**  **(2-7)** | **4.32**  **(2-9)** | **2.45**  **(2-7)** | **2.65**  **(2-7)** | **4.65**  **(2-9)** | **2.54**  **(2-7)** | **2.32**  **(2-7)** | **4.65**  **(2-9)** | **2.65**  **(2-7)** | **2.44**  **(2-7)** | **4.33**  **(2-9)** |
|  |  | Corpus | **3.73**  **(2-9)** | **4.23**  **(2-9)** | **6.54**  **(2-9)** | **3.65**  **(2-9)** | **4.98**  **(2-9)** | **6.87**  **(2-9)** | **3.65**  **(2-9)** | **4.34**  **(2-9)** | **6.76**  **(2-9)** | **3.66**  **(2-9)** | **4.76**  **(2-9)** | **6.77**  **(2-12)** |

| Gut hormone | H.pylori status | **Tissue isolation site** | **Gastric Cancer (GC)** | | | | | | | | | | | |
| --- | --- | --- | --- | --- | --- | --- | --- | --- | --- | --- | --- | --- | --- | --- |
|  |  |  | Neutrophils | | | Mononuclear cells | | | Atrophy | | | Intestinal metaplasia | | |
|  |  |  | Mild | Moderate | Severe | Mild | Moderate | Severe | Mild | Moderate | Severe | Mild | Moderate | Severe |
| Mean Plasma Leptin  ng/ml | *H. pylori* positive | Antrum | **4.87**  **(2-9)** | **3.44**  **(2-9)** | **6.49**  **(2-9)** | **4.54**  **(2-9)** | **3.33**  **(2-9)** | **6.71**  **(2-9)** | **4.54**  **(2-9)** | **3.56**  **(2-9)** | **6.71**  **(2-9)** | **4.65**  **(2-9)** | **3.66**  **(2-9)** | **6.67**  **(2-9)** |
|  |  | Corpus | **3.76**  **(2-9)** | **4.65**  **(2-9)** | **8.56**  **(5-14)** | **3.55**  **(2-9)** | **4.26**  **(2-9)** | **8.67**  **(5-14)** | **3.35**  **(2-9)** | **4.26**  **(2-9)** | **8.73**  **(5-14)** | **3.36**  **(2-9)** | **4.72**  **(2-9)** | **8.66**  **(5-14)** |
|  | *H. pylori* negative | Antrum | **2.66**  **(2-9)** | **2.48**  **(2-9)** | **4.65**  **(2-9)** | **2.87**  **(2-9)** | **2.69**  **(2-9)** | **4.43**  **(2-9)** | **2.21**  **(2-9)** | **2.75**  **(2-9)** | **4.77**  **(2-9)** | **2.24**  **(2-9)** | **2.67**  **(2-9)** | **4.87**  **(2-9)** |
|  |  | Corpus | **3.49**  **(2-9)** | **4.58**  **(2-9)** | **6.76**  **(2-9)** | **3.56**  **(2-9)** | **4.77**  **(2-9)** | **6.07**  **(2-9)** | **3.77**  **(2-9)** | **4.12**  **(2-9)** | **6.39**  **(2-9)** | **3.78**  **(2-9)** | **4.87**  **(2-9)** | **6.34**  **(2-9)** |

| Gut hormone | | *H. pylori* status | **Tissue isolation site** | **Nonulcer dyspepsia** **(NUD)** | | | | | | | | | | | |
| --- | --- | --- | --- | --- | --- | --- | --- | --- | --- | --- | --- | --- | --- | --- | --- |
|  |  |  |  | Neutrophils | | | Mononuclear cells | | | Atrophy | | | Intestinal metaplasia | | |
|  |  |  |  | Mild | Moderate | Severe | Mild | Moderate | Severe | Mild | Moderate | Severe | Mild | Moderate | Severe |
| Mean plasma Insulin mU/L | *H. pylori* positive | | Antrum | **27.5**  **(10-50)** | **32.4**  **(20-50)** | **29.7**  **(10-50)** | **34.9**  **(10-50)** | **37.5**  **(20-50)** | **44.0**  **(20-50)** | **23.7**  **(20-50)** | **19.5**  **(20-50)** | **25.6**  **(20-50)** | **20.3**  **(20-50)** | **26.1**  **(20-50)** | **23.9**  **(20-50)** |
|  |  |  | Corpus | **44.5**  **(20-75)** | **35.6**  **(20-50)** | **30.7**  **(20-50)** | **27.2**  **(20-50)** | **32.5**  **(20-50)** | **36.3**  **(20-50)** | **31.0**  **(20-50)** | **27.4**  **(20-50)** | **18.9**  **(20-50)** | **24.3**  **(20-50)** | **28.9**  **(20-50)** | **22.5**  **(20-50)** |
|  | *H. pylori* negative | | Antrum | **47.8**  **(20-80)** | **34.1**  **(20-75)** | **37.5**  **(20-80)** | **45.9**  **(20-80)** | **40.2**  **(20-80)** | **38.9**  **(20-80)** | **33.8**  **(20-70)** | **35.3**  **(20-70)** | **39.4**  **(20-80)** | **27.3**  **(20-50)** | **22.1**  **(20-50)** | **29.3**  **(20-50)** |
|  |  |  | Corpus | **40.4**  **(20-80)** | **42.9**  **(20-80)** | **36.8**  **(20-80)** | **42.5**  **(20-80)** | **38.4**  **(20-80)** | **28.5**  **(10-70)** | **22.5**  **(10-50)** | **28.1**  **(20-50)** | **17.9**  **(20-50)** | **24.9**  **(20-50)** | **27.5**  **(20-50)** | **23.9**  **(20-50)** |

| Gut hormone | | *H. pylori* status | **Tissue isolation site** | **Peptic Ulcer Disease (PUD)** | | | | | | | | | | | |
| --- | --- | --- | --- | --- | --- | --- | --- | --- | --- | --- | --- | --- | --- | --- | --- |
|  |  |  |  | Neutrophils | | | Mononuclear cells | | | Atrophy | | | Intestinal metaplasia | | |
|  |  |  |  | Mild | Moderate | Severe | Mild | Moderate | Severe | Mild | Moderate | Severe | Mild | Moderate | Severe |
| Mean plasma Insulin mU/L | *H. pylori* positive | | Antrum | **23.7**  **(20-50)** | **29.7**  **(20-50)** | **37.8**  **(20-50)** | **28.4**  **(20-50)** | **29.8**  **(20-50)** | **37.4**  **(20-50)** | **26.4**  **(20-50)** | **23.7**  **(20-50)** | **19.4**  **(20-50)** | **24.6**  **(20-50)** | **26.4**  **(20-50)** | **21.7**  **(20-50)** |
|  |  |  | Corpus | **36.3**  **(20-50)** | **31.0**  **(20-50)** | **27.4**  **(20-50)** | **18.9**  **(20-50)** | **24.3**  **(20-50)** | **28.9**  **(20-50)** | **45.9**  **(20-80)** | **40.2**  **(20-80)** | **38.9**  **(20-80)** | **33.8**  **(20-70)** | **35.3**  **(20-70)** | **39.4**  **(20-80)** |
|  | *H. pylori* negative | | Antrum | **37.5**  **(20-80)** | **45.9**  **(20-80)** | **40.2**  **(20-80)** | **38.9**  **(20-80)** | **33.8**  **(20-70)** | **28.1**  **(20-50)** | **17.9**  **(20-50)** | **24.9**  **(20-50)** | **27.5**  **(20-50)** | **28.1**  **(20-50)** | **38.4**  **(20-80)** | **28.5**  **(10-70)** |
|  |  |  | Corpus | **38.4**  **(20-80)** | **28.5**  **(10-70)** | **22.5**  **(10-50)** | **28.1**  **(20-50)** | **17.9**  **(20-50)** | **24.9**  **(20-50)** | **38.4**  **(20-80)** | **37.5**  **(20-80)** | **45.9**  **(20-80)** | **40.2**  **(20-80)** | **38.9**  **(20-80)** | **37.5**  **(20-80)** |

| Gut hormone | | *H. pylori* status | **Tissue isolation site** | **Gastric Cancer (GC)** | | | | | | | | | | | |
| --- | --- | --- | --- | --- | --- | --- | --- | --- | --- | --- | --- | --- | --- | --- | --- |
|  |  |  |  | Neutrophils | | | Mononuclear cells | | | Atrophy | | | Intestinal metaplasia | | |
|  |  |  |  | Mild | Moderate | Severe | Mild | Moderate | Severe | Mild | Moderate | Severe | Mild | Moderate | Severe |
| Mean plasma Insulin mU/L | *H. pylori* positive | | Antrum | **27.2**  **(20-50)** | **32.5**  **(20-50)** | **36.3**  **(20-50)** | **31.0**  **(20-50)** | **27.4**  **(20-50)** | **18.9**  **(20-50)** | **24.3**  **(20-50)** | **28.9**  **(20-50)** | **27.2**  **(20-50)** | **35.3**  **(20-70)** | **39.4**  **(20-80)** | **27.3**  **(20-50)** |
|  |  |  | Corpus | **(20-50)** | **(20-50)** | **(20-50)** | **(20-50)** | **(20-50)** | **(20-50)** | **(20-50)** | **(20-50)** | **(20-50)** | **(20-50)** | **(20-50)** | **(20-50)** |
|  | *H. pylori* negative | | Antrum | **38.9**  **(20-80)** | **33.8**  **(20-70)** | **28.1**  **(20-50)** | **17.9**  **(20-50)** | **24.9**  **(20-50)** | **27.5**  **(20-50)** | **34.1**  **(20-75)** | **37.5**  **(20-80)** | **45.9**  **(20-80)** | **40.2**  **(20-80)** | **38.9**  **(20-80)** | **33.8**  **(20-70)** |
|  |  |  | Corpus | **36.8**  **(20-80)** | **42.5**  **(20-80)** | **38.4**  **(20-80)** | **28.5**  **(10-70)** | **22.5**  **(10-50)** | **28.1**  **(20-50)** | **32.5**  **(20-75)** | **38.5**  **(20-80)** | **41.9**  **(20-80)** | **45.2**  **(20-80)** | **32.9**  **(20-80)** | **29.1**  **(20-75)** |
| Peptide protein | *H. pylori* status | | **Tissue isolation site** | **Nonulcer dyspepsia** **(NUD)** | | | | | | | | | | | |
|  |  |  |  | Neutrophils | | | Mononuclear cells | | | Atrophy | | | Intestinal metaplasia | | |
|  |  |  |  | Mild | Moderate | Severe | Mild | Moderate | Severe | Mild | Moderate | Severe | Mild | Moderate | Severe |
| Mean plasma Neuropeptide Y  ng/L | *H. pylori* positive | | Antrum | **374.2**  **(300-400)** | **359.5**  **(300-400)** | **333.3**  **(300-400)** | **316.7**  **(300-400)** | **319.8**  **(300-400)** | **306.3**  **(300-400)** | **315.5**  **(300-400)** | **310.7**  **(300-400)** | **318.8**  **(300-400)** | **327.3**  **(300-400)** | **316.8**  **(300-400)** | **310.7**  **(300-400)** |
|  |  |  | Corpus | **364.2**  **(300-400)** | **351.7**  **(300-400)** | **342.4**  **(300-400)** | **344.8**  **(300-400)** | **323.5**  **(300-400)** | **335.4**  **(300-400)** | **340.5**  **(300-400)** | **321.7**  **(300-400)** | **338.9**  **(300-400)** | **320.4**  **(300-400)** | **347.4**  **(300-400)** | **312.2**  **(300-400)** |
|  | *H. pylori* negative | | Antrum | **393.6**  **(300-400)** | **381.8**  **(300-400)** | **342.5**  **(300-400)** | **337.4**  **(300-400)** | **349.1**  **(300-400)** | **332.8**  **(300-400)** | **347.9**  **(300-400)** | **341.0**  **(300-400)** | **345.8**  **(300-400)** | **334.0**  **(300-400)** | **316.5**  **(300-400)** | **327.4**  **(300-400)** |
|  |  |  | Corpus | **364.5**  **(300-400)** | **379.2**  **(300-400)** | **347.5**  **(300-400)** | **321.0**  **(300-400)** | **344.5**  **(300-400)** | **361.7**  **(300-400)** | **325.6**  **(300-400)** | **342.0**  **(300-400)** | **357.5**  **(300-400)** | **344.1**  **(300-400)** | **356.7**  **(300-400)** | **342.8**  **(300-400)** |

| Peptide protein | *H. pylori* status | **Tissue isolation site** | **Peptic Ulcer Disease (PUD)** | | | | | | | | | | | |
| --- | --- | --- | --- | --- | --- | --- | --- | --- | --- | --- | --- | --- | --- | --- |
|  |  |  | Neutrophils | | | Mononuclear cells | | | Atrophy | | | Intestinal metaplasia | | |
|  |  |  | Mild | Moderate | Severe | Mild | Moderate | Severe | Mild | Moderate | Severe | Mild | Moderate | Severe |
| Mean plasma Neuropeptide Y  ng/L | *H. pylori* positive | Antrum | **323.5**  **(300-400)** | **335.4**  **(300-400)** | **340.5**  **(300-400)** | **321.7**  **(300-400)** | **323.5**  **(300-400)** | **315.5**  **(300-400)** | **310.7**  **(300-400)** | **318.8**  **(300-400)** | **327.3**  **(300-400)** | **315.5**  **(300-400)** | **332.7**  **(300-400)** | **337.4**  **(300-400** |
|  |  | Corpus | **316.7**  **(300-400)** | **319.2**  **(300-400)** | **306.3**  **(300-400)** | **315.3**  **(300-400)** | **310.6**  **(300-400)** | **316.7**  **(300-400)** | **346.7**  **(300-400)** | **329.8**  **(300-400)** | **376.3**  **(300-400)** | **325.5**  **(300-400)** | **310.7**  **(300-400)** | **366.7**  **(300-400)** |
|  | *H. pylori* negative | Antrum | **344.5**  **(300-400)** | **361.7**  **(300-400)** | **325.6**  **(300-400)** | **342.0**  **(300-400)** | **357.9**  **(300-400)** | **344.2**  **(300-400)** | **342.7**  **(300-400)** | **337.1**  **(300-400)** | **349.6**  **(300-400)** | **332.2**  **(300-400)** | **347.4**  **(300-400)** | **342.6**  **(300-400)** |
|  |  | Corpus | **326.0**  **(300-400)** | **364.5**  **(300-400)** | **341.7**  **(300-400)** | **331.6**  **(300-400)** | **352.0**  **(300-400)** | **367.5**  **(300-400)** | **329.0**  **(300-400)** | **348.5**  **(300-400)** | **362.7**  **(300-400)** | **325.7**  **(300-400)** | **342.0**  **(300-400)** | **357.5**  **(300-400)** |

| Peptide protein | *H. pylori* status | **Tissue isolation site** | **Gastric Cancer (GC)** | | | | | | | | | | | |
| --- | --- | --- | --- | --- | --- | --- | --- | --- | --- | --- | --- | --- | --- | --- |
|  |  |  | Neutrophils | | | Mononuclear cells | | | Atrophy | | | Intestinal metaplasia | | |
|  |  |  | Mild | Moderate | Severe | Mild | Moderate | Severe | Mild | Moderate | Severe | Mild | Moderate | Severe |
| Mean plasma Neuropeptide Y  ng/L | *H. pylori* positive | Antrum | **323.4**  **(300-400)** | **335.8**  **(300-400)** | **340.2**  **(300-400)** | **321.6**  **(300-400)** | **338.9**  **(300-400)** | **323.5**  **(300-400)** | **339.4**  **(300-400)** | **355.5**  **(300-400)** | **329.7**  **(300-400)** | **358.9**  **(300-400)** | **320.4**  **(300-400)** | **327.4**  **(300-400)** |
|  |  | Corpus | **343.7**  **(300-400)** | **352.4**  **(300-400)** | **354.8**  **(300-400)** | **333.5**  **(300-400)** | **330.4**  **(300-400)** | **343.5**  **(300-400)** | **306.3**  **(300-400)** | **318.3**  **(300-400)** | **310.6**  **(300-400)** | **319.7**  **(300-400)** | **366.7**  **(300-400)** | **359.8**  **(300-400)** |
|  | *H. pylori* negative | Antrum | **342.0**  **(300-400)** | **357.9**  **(300-400)** | **344.2**  **(300-400)** | **342.7**  **(300-400)** | **337.1**  **(300-400)** | **349.6**  **(300-400)** | **332.2**  **(300-400)** | **341.0**  **(300-400)** | **345.8**  **(300-400)** | **334.0**  **(300-400)** | **316.5**  **(300-400)** | **327.4**  **(300-400)** |
|  |  | Corpus | **342.4**  **(300-400)** | **344.8**  **(300-400)** | **323.5**  **(300-400)** | **335.4**  **(300-400)** | **340.5**  **(300-400)** | **342.0**  **(300-400)** | **357.5**  **(300-400)** | **344.1**  **(300-400)** | **335.4**  **(300-400)** | **340.5**  **(300-400)** | **321.7**  **(300-400)** | **338.9**  **(300-400)** |

| Inflammatory cytokine | *H. pylori* status | **Tissue isolation site** | **Nonulcer dyspepsia** **(NUD)** | | | | | | | | | | | |
| --- | --- | --- | --- | --- | --- | --- | --- | --- | --- | --- | --- | --- | --- | --- |
|  |  |  | Neutrophils | | | Mononuclear cells | | | Atrophy | | | Intestinal metaplasia | | |
|  |  |  | Mild | Moderate | Severe | Mild | Moderate | Severe | Mild | Moderate | Severe | Mild | Moderate | Severe |
| Mean plasma IFN- gamma ng/L | *H. pylori* positive | Antrum | **44.8**  **(10-100)** | **152.0**  **(50-200)** | **160.6**  **(100-200)** | **57.9**  **(40-100)** | **120.5**  **(50-150)** | **175.3**  **(100-200)** | **143.2**  **(100-200)** | **164.5**  **(100-200)** | **159.7**  **(100-200)** | **133.2**  **(100-200)** | **146.5**  **(100-200)** | **159.3**  **(100-200)** |
|  |  | Corpus | **56.4**  **(10-100)** | **141.7**  **(50-200)** | **174.7**  **(100-200)** | **69.2**  **(40-100)** | **148.3**  **(50-150)** | **182.3**  **(100-200)** | **155.8**  **(100-200)** | **173.9**  **(100-200)** | **185.9**  **(100-200)** | **172.8**  **(100-200)** | **159.3**  **(100-200)** | **166.7**  **(100-200)** |
|  | *H. pylori* negative | Antrum | **62.0**  **(10-100)** | **155.3**  **(50-200)** | **147.6**  **(100-200)** | **78.7**  **(40-100)** | **131.6**  **(50-150)** | **166.9**  **(100-200)** | **134.5**  **(100-200)** | **164.2**  **(100-200)** | **171.7**  **(100-200)** | **163.3**  **(100-200)** | **137.5**  **(100-200)** | **149.6**  **(100-200)** |
|  |  | Corpus | **66.7**  **(10-100)** | **143.8**  **(50-200)** | **131.4**  **(100-200)** | **89.9**  **(40-100)** | **128.3**  **(50-150)** | **152.9**  **(100-200)** | **148.6**  **(100-200)** | **152.5**  **(100-200)** | **169.2**  **(100-200)** | **179.3**  **(100-250)** | **128.6**  **(100-200)** | **158.5**  **(100-200)** |

| Inflammatory cytokine | *H. pylori* status | **Tissue isolation site** | **Peptic Ulcer Disease (PUD)** | | | | | | | | | | | |
| --- | --- | --- | --- | --- | --- | --- | --- | --- | --- | --- | --- | --- | --- | --- |
|  |  |  | Neutrophils | | | Mononuclear cells | | | Atrophy | | | Intestinal metaplasia | | |
|  |  |  | Mild | Moderate | Severe | Mild | Moderate | Severe | Mild | Moderate | Severe | Mild | Moderate | Severe |
| Mean plasma IFN- gamma ng/L | *H. pylori* positive | Antrum | **57.3**  **(40-100)** | **120.6**  **(50-150)** | **174.3**  **(100-200)** | **133.2**  **(100-200)** | **154.5**  **(100-200)** | **169.7**  **(100-200)** | **133.2**  **(100-200)** | **148.3**  **(50-150)** | **182.3**  **(100-200)** | **155.8**  **(100-200)** | **173.9**  **(100-200)** | **185.9**  **(100-200)** |
|  |  | Corpus | **67.2**  **(40-100)** | **148.3**  **(50-150)** | **182.3**  **(100-200)** | **155.8**  **(100-200)** | **173.9**  **(100-200)** | **62.7**  **(40-100)** | **182.3**  **(100-200)** | **159.8**  **(100-200)** | **173.9**  **(100-200)** | **181.9**  **(100-200)** | **186.3**  **(100-200)** | **173.9**  **(100-200)** |
|  | *H. pylori* negative | Antrum | **65.9**  **(100-200)** | **134.5**  **(100-200)** | **164.2**  **(100-200)** | **171.7**  **(100-200)** | **163.3**  **(100-200)** | **137.5**  **(100-200)** | **159.6**  **(100-200)** | **131.6**  **(50-150)** | **168.9**  **(100-200)** | **134.5**  **(100-200)** | **154.2**  **(100-200)** | **133.6**  **(50-150)** |
|  |  | Corpus | **83.9**  **(40-100)** | **138.3**  **(50-150)** | **142.9**  **(100-200)** | **158.6**  **(100-200)** | **162.5**  **(100-200)** | **169.2**  **(100-200)** | **149.3**  **(100-250)** | **128.9**  **(50-150)** | **155.8**  **(100-200)** | **173.9**  **(100-200)** | **162.7**  **(40-100)** | **182.3**  **(100-200)** |

| Inflammatory cytokine | | *H. pylori* status | **Tissue isolation site** | **Gastric Cancer (GC)** | | | | | | | | | | | |
| --- | --- | --- | --- | --- | --- | --- | --- | --- | --- | --- | --- | --- | --- | --- | --- |
|  |  |  |  | Neutrophils | | | Mononuclear cells | | | Atrophy | | | Intestinal metaplasia | | |
|  |  |  |  | Mild | Moderate | Severe | Mild | Moderate | Severe | Mild | Moderate | Severe | Mild | Moderate | Severe |
| Mean plasma IFN- gamma ng/L | | *H. pylori* positive | Antrum | **77.3**  **(40-100)** | **130.6**  **(50-150)** | **154.4**  **(100-200)** | **143.6**  **(100-200)** | **154.5**  **(100-200)** | **149.7**  **(100-200)** | **153.2**  **(100-200)** | **158.3**  **(50-150)** | **172.3**  **(100-200)** | **162.8**  **(100-200)** | **171.4**  **(100-200)** | **182.9**  **(100-200)** |
|  |  |  | Corpus | **58.8**  **(100-200)** | **173.9**  **(100-200)** | **162.7**  **(40-100)** | **182.3**  **(100-200)** | **145.8**  **(100-200)** | **155.8**  **(100-200)** | **145.8**  **(100-200)** | **153.9**  **(100-200)** | **162.7**  **(40-100)** | **182.3**  **(100-200)** | **175.9**  **(100-200)** | **186.0**  **(100-200)** |
|  |  | *H. pylori* negative | Antrum | **89.0**  **(40-150)** | **143.6**  **(100-200)** | **128.3**  **(50-150)** | **152.9**  **(100-200)** | **148.6**  **(100-200)** | **152.5**  **(100-200)** | **169.2**  **(100-200)** | **179.3**  **(100-250)** | **128.6**  **(100-200)** | **131.4**  **(100-200)** | **89.9**  **(40-100)** | **128.3**  **(50-150)** |
|  |  |  | Corpus | **71.7**  **(100-150)** | **163.3**  **(100-200)** | **137.5**  **(100-200)** | **159.6**  **(100-200)** | **131.6**  **(50-150)** | **168.9**  **(100-200)** | **171.7**  **(100-200)** | **163.3**  **(100-200)** | **137.5**  **(100-200)** | **159.6**  **(100-200)** | **131.6**  **(50-150)** | **168.9**  **(100-200)** |
| Inflammatory cytokine | | *H. pylori* status | **Tissue isolation site** | **Nonulcer dyspepsia** **(NUD)** | | | | | | | | | | | |
|  |  |  |  | Neutrophils | | | Mononuclear cells | | | Atrophy | | | Intestinal metaplasia | | |
|  |  |  |  | Mild | Moderate | Severe | Mild | Moderate | Severe | Mild | Moderate | Severe | Mild | Moderate | Severe |
| Mean plasma TNF - alpha  ng/L | | *H. pylori* positive | Antrum | **133.4**  **(75-200)** | **140.2**  **(75-200)** | **476.7**  **(200-500)** | **466.1**  **(200-600)** | **549.7**  **(200-600)** | **704.2**  **(400-800)** | **630.3**  **(400-700)** | **726.5**  **(500-800)** | **809.6**  **(600-900)** | **714.3**  **(600-800)** | **765.7**  **(600-800)** | **814.2**  **(600-900)** |
|  |  |  | Corpus | **126.4**  **(75-200)** | **153.5**  **(75-200)** | **435.8**  **(200-500)** | **459.8**  **(200-600)** | **512.9**  **(200-600)** | **727.4**  **(400-800)** | **589.9**  **(400-700)** | **739.6**  **(500-800)** | **811.3**  **(600-900)** | **732.3**  **(600-800)** | **769.8**  **(600-800)** | **807.7**  **(600-900)** |
|  |  | *H. pylori* negative | Antrum | **148.7**  **(75-200)** | **163.2**  **(75-200)** | **486.8**  **(200-500)** | **497.1**  **(200-600)** | **559.7**  **(200-600)** | **709.1**  **(400-800)** | **646.3**  **(400-700)** | **756.5**  **(500-800)** | **819.6**  **(600-900)** | **722.9**  **(600-800)** | **779.7**  **(600-800)** | **834.0 (600-900)** |
|  |  |  | Corpus | **136.9**  **(75-200)** | **149.5**  **(75-200)** | **448.8**  **(200-500)** | **467.8**  **(200-600)** | **533.9**  **(200-600)** | **736.4**  **(400-800)** | **590.9**  **(400-700)** | **769.6**  **(500-800)** | **823.3**  **(600-900)** | **742.3**  **(600-800)** | **786.8**  **(600-800)** | **817.7**  **(600-900)** |

| Inflammatory cytokine | *H. pylori* status | **Tissue isolation site** | **Peptic Ulcer Disease (PUD)** | | | | | | | | | | | |
| --- | --- | --- | --- | --- | --- | --- | --- | --- | --- | --- | --- | --- | --- | --- |
|  |  |  | Neutrophils | | | Mononuclear cells | | | Atrophy | | | Intestinal metaplasia | | |
|  |  |  | Mild | Moderate | Severe | Mild | Moderate | Severe | Mild | Moderate | Severe | Mild | Moderate | Severe |
| Mean plasma TNF - alpha  ng/L | *H. pylori* positive | Antrum | **128.8**  **(75-200)** | **147.2**  **(75-200)** | **451.7**  **(200-500)** | **469.8**  **(200-600)** | **559.4**  **(200-600)** | **718.2**  **(400-800)** | **639.8**  **(400-700)** | **733.9**  **(500-800)** | **816.9**  **(600-900)** | **723.8**  **(600-800)** | **772.2**  **(600-800)** | **827.8**  **(600-900)** |
|  |  | Corpus | **143.5**  **(75-200)** | **168.5**  **(75-200)** | **449.5**  **(200-500)** | **466.4**  **(200-600)** | **576.9**  **(200-600)** | **729.4**  **(400-800)** | **599.8**  **(400-700)** | **747.6**  **(500-800)** | **821.8**  **(600-900)** | **748.1**  **(600-800)** | **792.1**  **(600-800)** | **815.9**  **(600-900)** |
|  | *H. pylori* negative | Antrum | **137.9**  **(75-200)** | **151.7**  **(75-200)** | **477.7**  **(200-500)** | **478.2**  **(200-600)** | **559.4**  **(200-600)** | **727.4**  **(400-800)** | **589.9**  **(400-700)** | **739.6**  **(500-800)** | **811.3**  **(600-900)** | **727.4**  **(400-800)** | **779.7**  **(600-800)** | **822.0 (600-900)** |
|  |  | Corpus | **154.8**  **(75-200)** | **171.5**  **(75-200)** | **449.5**  **(200-500)** | **465.8**  **(200-600)** | **589.9**  **(200-600)** | **589.9**  **(400-700)** | **739.6**  **(500-800)** | **811.3**  **(600-900)** | **732.3**  **(600-800)** | **769.8**  **(600-800)** | **765.7**  **(600-800)** | **810.2**  **(600-900)** |

| Inflammatory cytokine | *H. pylori* status | **Tissue isolation site** | **Gastric Cancer (GC)** | | | | | | | | | | | |
| --- | --- | --- | --- | --- | --- | --- | --- | --- | --- | --- | --- | --- | --- | --- |
|  |  |  | Neutrophils | | | Mononuclear cells | | | Atrophy | | | Intestinal metaplasia | | |
|  |  |  | Mild | Moderate | Severe | Mild | Moderate | Severe | Mild | Moderate | Severe | Mild | Moderate | Severe |
| Mean plasma TNF - alpha  ng/L | *H. pylori* positive | Antrum | **138.4**  **(75-200)** | **163.8**  **(75-200)** | **449.8**  **(200-500)** | **465.8**  **(200-600)** | **528.9**  **(200-600)** | **735.4**  **(400-800)** | **592.9**  **(400-700)** | **759.1**  **(500-800)** | **827.3**  **(600-900)** | **729.8**  **(600-800)** | **788.1**  **(600-800)** | **828.2**  **(600-900)** |
|  |  | Corpus | **141.8**  **(75-200)** | **171.8**  **(75-200)** | **576.9**  **(200-600)** | **429.4**  **(400-800)** | **549.8**  **(400-700)** | **747.6**  **(500-800)** | **521.8**  **(600-900)** | **748.4**  **(600-800)** | **819.6**  **(600-900)** | **722.9**  **(600-800)** | **779.7**  **(600-800)** | **834.0 (600-900)** |
|  | *H. pylori* negative | Antrum | **139.1**  **(75-200)** | **184.8**  **(75-200)** | **727.4**  **(400-800)** | **589.9**  **(400-700)** | **739.6**  **(500-800)** | **811.3**  **(600-900)** | **727.4**  **(400-800)** | **727.4**  **(400-800)** | **756.5**  **(500-800)** | **819.6**  **(600-900)** | **722.9**  **(600-800)** | **756.5**  **(500-800)** |
|  |  | Corpus | **133.4**  **(75-200)** | **175.8**  **(75-200)** | **718.2**  **(400-800)** | **639.8**  **(400-700)** | **733.9**  **(500-800)** | **816.9**  **(600-900)** | **723.8**  **(600-800)** | **718.2**  **(400-800)** | **742.3**  **(600-800)** | **786.8**  **(600-800)** | **817.7**  **(600-900)** | **742.3**  **(600-800)** |

| Inflammatory cytokine | H. pylori status | **Tissue isolation site** | **Nonulcer dyspepsia** **(NUD)** | | | | | | | | | | | |
| --- | --- | --- | --- | --- | --- | --- | --- | --- | --- | --- | --- | --- | --- | --- |
|  |  |  | Neutrophils | | | Mononuclear cells | | | Atrophy | | | Intestinal metaplasia | | |
|  |  |  | Mild | Moderate | Severe | Mild | Moderate | Severe | Mild | Moderate | Severe | Mild | Moderate | Severe |
| Mean plasma TNFR - 2  Pg/ml | H. pylori positive | Antrum | **63.8**  **(20-100)** | **73.6**  **(20-100)** | **168.7**  **(50-200)** | **206.3**  **(100-300)** | **261.2**  **(100-200)** | **309.6**  **(100-400)** | **514.5**  **(200-600)** | **574.6**  **(200-700)** | **592.6**  **(200-700)** | **507.4**  **(200-700)** | **556.5**  **(200-700)** | **597.3**  **(200-700)** |
|  |  | Corpus | **72.6**  **(20-100)** | **85.2**  **(20-100)** | **173.8**  **(50-200)** | **227.8**  **(100-300)** | **274.8**  **(100-200)** | **314.7**  **(100-400)** | **493.9**  **(200-600)** | **582.1**  **(200-700)** | **590.9**  **(200-700)** | **515.8**  **(200-700)** | **568.5**  **(200-700)** | **582.3**  **(200-700)** |
|  | H. pylori negative | Antrum | **34.0**  **(20-100)** | **62.9**  **(20-100)** | **132.3**  **(50-200)** | **165.2**  **(100-200)** | **246.7**  **(100-200)** | **299.8**  **(100-300)** | **247.3**  **(200-600)** | **263.3**  **(200-700)** | **294.2**  **(200-700)** | **251.2**  **(200-700)** | **346.5**  **(200-700)** | **521.7**  **(200-700)** |
|  |  | Corpus | **78.5**  **(20-100)** | **89.4**  **(20-100)** | **153.9**  **(50-200)** | **237.5**  **(100-300)** | **286.4**  **(100-200)** | **327.3**  **(100-400)** | **521.3**  **(200-600)** | **576.8**  **(200-700)** | **589.5**  **(200-700)** | **525.3**  **(200-700)** | **576.5**  **(200-700)** | **592.8**  **(200-700)** |

| Inflammatory cytokine | *H. pylori* status | **Tissue isolation site** | **Peptic Ulcer Disease (PUD)** | | | | | | | | | | | |
| --- | --- | --- | --- | --- | --- | --- | --- | --- | --- | --- | --- | --- | --- | --- |
|  |  |  | Neutrophils | | | Mononuclear cells | | | Atrophy | | | Intestinal metaplasia | | |
|  |  |  | Mild | Moderate | Severe | Mild | Moderate | Severe | Mild | Moderate | Severe | Mild | Moderate | Severe |
| Mean plasma TNFR - 2  Pg/ml | *H. pylori* positive | Antrum | **69.7**  **(20-100)** | **77.3**  **(20-100)** | **179.6**  **(50-200)** | **217.3**  **(100-300)** | **275.2**  **(100-200)** | **326.6**  **(100-400)** | **527.5**  **(200-600)** | **582.8**  **(200-700)** | **584.7**  **(200-700)** | **523.8**  **(200-700)** | **568.9**  **(200-700)** | **586.6**  **(200-700)** |
|  |  | Corpus | **77.3**  **(20-100)** | **88.9**  **(20-100)** | **182.8**  **(50-200)** | **242.8**  **(100-300)** | **281.0**  **(100-200)** | **328.0**  **(100-400)** | **468.8**  **(200-600)** | **592.5**  **(200-700)** | **583.8**  **(200-700)** | **522.7**  **(200-700)** | **571.4**  **(200-700)** | **594.5**  **(200-700)** |
|  | *H. pylori* negative | Antrum | **45.0**  **(20-100)** | **67.5**  **(20-100)** | **144.7**  **(50-200)** | **299.8**  **(100-300)** | **247.3**  **(200-600)** | **263.3**  **(200-700)** | **294.2**  **(200-700)** | **263.3**  **(200-700)** | **294.2**  **(200-700)** | **251.2**  **(200-700)** | **348.2**  **(200-700)** | **538.7**  **(200-700)** |
|  |  | Corpus | **87.3**  **(20-100)** | **93.3**  **(20-100)** | **161.6**  **(50-200)** | **246.7**  **(100-200)** | **299.8**  **(100-300)** | **247.3**  **(200-600)** | **263.3**  **(200-700)** | **294.2**  **(200-700)** | **244.8**  **(100-200)** | **525.3**  **(200-700)** | **572.7**  **(200-700)** | **597.1**  **(200-700)** |

| Inflammatory cytokine | *H. pylori* status | **Tissue isolation site** | **Gastric Cancer (GC)** | | | | | | | | | | | |
| --- | --- | --- | --- | --- | --- | --- | --- | --- | --- | --- | --- | --- | --- | --- |
|  |  |  | Neutrophils | | | Mononuclear cells | | | Atrophy | | | Intestinal metaplasia | | |
|  |  |  | Mild | Moderate | Severe | Mild | Moderate | Severe | Mild | Moderate | Severe | Mild | Moderate | Severe |
| Mean plasma TNFR - 2  Pg/ml | *H. pylori* positive | Antrum | **72.6**  **(20-100)** | **75.6**  **(20-100)** | **188.7**  **(50-200)** | **222.3**  **(100-300)** | **273.2**  **(100-200)** | **315.6**  **(100-400)** | **520.5**  **(200-600)** | **583.6**  **(200-700)** | **584.7**  **(200-700)** | **512.8**  **(200-700)** | **561.5**  **(200-700)** | **590.3**  **(200-700)** |
|  |  | Corpus | **79.7**  **(20-100)** | **87.2**  **(20-100)** | **183.8**  **(50-200)** | **231.8**  **(100-300)** | **287.8**  **(100-200)** | **327.7**  **(100-400)** | **483.7**  **(200-600)** | **597.1**  **(200-700)** | **583.0**  **(200-700)** | **527.8**  **(200-700)** | **574.5**  **(200-700)** | **593.3**  **(200-700)** |
|  | *H. pylori* negative | Antrum | **49.4**  **(20-100)** | **68.9**  **(20-100)** | **147.3**  **(50-200)** | **174.2**  **(100-200)** | **252.7**  **(100-200)** | **310.8**  **(100-300)** | **277.3**  **(200-600)** | **274.3**  **(200-700)** | **571.9**  **(200-700)** | **520.2**  **(200-700)** | **552.5**  **(200-700)** | **548.7**  **(200-700)** |
|  |  | Corpus | **85.8**  **(20-100)** | **81.4**  **(20-100)** | **167.7**  **(50-200)** | **245.5**  **(100-300)** | **278.4**  **(100-200)** | **338.3**  **(100-400)** | **548.3**  **(200-600)** | **571.5**  **(200-700)** | **579.3**  **(200-700)** | **537.3**  **(200-700)** | **580.5**  **(200-700)** | **588.8**  **(200-700)** |

| Inflammatory cytokine | *H. pylori* status | **Tissue isolation site** | **Nonulcer dyspepsia** **(NUD)** | | | | | | | | | | | |
| --- | --- | --- | --- | --- | --- | --- | --- | --- | --- | --- | --- | --- | --- | --- |
|  |  |  | Neutrophils | | | Mononuclear cells | | | Atrophy | | | Intestinal metaplasia | | |
|  |  |  | Mild | Moderate | Severe | Mild | Moderate | Severe | Mild | Moderate | Severe | Mild | Moderate | Severe |
| Mean plasma Interleukins - 4  pg/ml | *H. pylori* positive | Antrum | **31.1**  **(20-100)** | **59.5**  **(20-100)** | **121.7**  **(100-200)** | **190.6**  **(100-250)** | **263.1**  **(100-300)** | **275.5**  **(100-300)** | **210.7**  **(100-300)** | **245.3**  **(200-400)** | **293.5**  **(200-400)** | **237.5**  **(200-300)** | **259.5**  **(200-400)** | **278.3**  **(200-400)** |
|  |  | Corpus | **42.7**  **(20-100)** | **62.4**  **(20-100)** | **134.8**  **(100-200)** | **182.8**  **(100-250)** | **275.8**  **(100-300)** | **281.5**  **(100-300)** | **225.5**  **(100-300)** | **257.8**  **(200-400)** | **309.7**  **(200-400)** | **241.5**  **(200-300)** | **266.9**  **(200-400)** | **311.8**  **(200-400)** |
|  | *H. pylori* negative | Antrum | **54.8**  **(20-100)** | **72.5**  **(20-100)** | **142.4**  **(100-200)** | **172.9**  **(100-250)** | **258.3**  **(100-300)** | **274.0**  **(100-300)** | **227.9**  **(100-300)** | **265.5**  **(200-400)** | **315.3**  **(200-400)** | **256.5**  **(200-300)** | **272.7**  **(200-400)** | **324.6**  **(200-400)** |
|  |  | Corpus | **47.6**  **(20-100)** | **79.7**  **(20-100)** | **157.5**  **(100-200)** | **197.4**  **(100-250)** | **247.9**  **(100-300)** | **288.3**  **(100-300)** | **232.6**  **(100-300)** | **268.3**  **(200-400)** | **339.8**  **(200-400)** | **262.5**  **(200-300)** | **288.4**  **(200-400)** | **327.7**  **(200-400)** |

| Inflammatory cytokine | *H. pylori* status | **Tissue isolation site** | **Peptic Ulcer Disease (PUD)** | | | | | | | | | | | |
| --- | --- | --- | --- | --- | --- | --- | --- | --- | --- | --- | --- | --- | --- | --- |
|  |  |  | Neutrophils | | | Mononuclear cells | | | Atrophy | | | Intestinal metaplasia | | |
|  |  |  | Mild | Moderate | Severe | Mild | Moderate | Severe | Mild | Moderate | Severe | Mild | Moderate | Severe |
| Mean plasma Interleukins - 4  pg/ml | *H. pylori* positive | Antrum | **34.8**  **(20-100)** | **62.5**  **(20-100)** | **134.7**  **(100-200)** | **196.6**  **(100-250)** | **278.1**  **(100-300)** | **282.5**  **(100-300)** | **216.7**  **(100-300)** | **251.3**  **(200-400)** | **305.5**  **(200-400)** | **246.5**  **(200-300)** | **265.5**  **(200-400)** | **284.3**  **(200-400)** |
|  |  | Corpus | **48.5**  **(20-100)** | **67.4**  **(20-100)** | **139.8**  **(100-200)** | **195.8**  **(100-250)** | **286.8**  **(100-300)** | **289.5**  **(100-300)** | **234.5**  **(100-300)** | **262.8**  **(200-400)** | **322.7**  **(200-400)** | **253.5**  **(200-300)** | **275.9**  **(200-400)** | **325.8**  **(200-400)** |
|  | *H. pylori* negative | Antrum | **59.4**  **(20-100)** | **82.5**  **(20-100)** | **151.4**  **(100-200)** | **187.9**  **(100-250)** | **266.3**  **(100-300)** | **283.0**  **(100-300)** | **239.9**  **(100-300)** | **273.5**  **(200-400)** | **325.3**  **(200-400)** | **264.5**  **(200-300)** | **283.7**  **(200-400)** | **336.6**  **(200-400)** |
|  |  | Corpus | **56.2**  **(20-100)** | **85.7**  **(20-100)** | **164.5**  **(100-200)** | **207.4**  **(100-250)** | **253.9**  **(100-300)** | **296.3**  **(100-300)** | **245.6**  **(100-300)** | **276.3**  **(200-400)** | **348.9**  **(200-400)** | **271.5**  **(200-300)** | **297.4**  **(200-400)** | **345.7**  **(200-400)** |

| Inflammatory cytokine | *H. pylori* status | **Tissue isolation site** | **Gastric Cancer (GC)** | | | | | | | | | | | |
| --- | --- | --- | --- | --- | --- | --- | --- | --- | --- | --- | --- | --- | --- | --- |
|  |  |  | Neutrophils | | | Mononuclear cells | | | Atrophy | | | Intestinal metaplasia | | |
|  |  |  | Mild | Moderate | Severe | Mild | Moderate | Severe | Mild | Moderate | Severe | Mild | Moderate | Severe |
| Mean plasma Interleukins - 4  pg/ml | *H. pylori* positive | Antrum | **43.6**  **(20-100)** | **67.5**  **(20-100)** | **142.7**  **(100-200)** | **205.6**  **(100-250)** | **263.1**  **(100-300)** | **288.5**  **(100-300)** | **223.7**  **(100-300)** | **262.3**  **(200-400)** | **314.5**  **(200-400)** | **257.5**  **(200-300)** | **274.5**  **(200-400)** | **292.3**  **(200-400)** |
|  |  | Corpus | **54.5**  **(20-100)** | **72.4**  **(20-100)** | **145.8**  **(100-200)** | **209.8**  **(100-250)** | **292.8**  **(100-300)** | **294.5**  **(100-300)** | **246.5**  **(100-300)** | **268.8**  **(200-400)** | **336.7**  **(200-400)** | **266.5**  **(200-300)** | **289.9**  **(200-400)** | **356.8**  **(200-400)** |
|  | *H. pylori* negative | Antrum | **62.4**  **(20-100)** | **89.5**  **(20-100)** | **159.4**  **(100-200)** | **203.9**  **(100-250)** | **272.3**  **(100-300)** | **298.0**  **(100-300)** | **249.9**  **(100-300)** | **284.5**  **(200-400)** | **339.8**  **(200-400)** | **275.5**  **(200-300)** | **292.7**  **(200-400)** | **357.6**  **(200-400)** |
|  |  | Corpus | **67.9**  **(20-100)** | **92.7**  **(20-100)** | **168.5**  **(100-200)** | **215.4**  **(100-250)** | **267.9**  **(100-300)** | **305.3**  **(100-300)** | **253.6**  **(100-300)** | **287.3**  **(200-400)** | **357.9**  **(200-400)** | **289.5**  **(200-300)** | **314.4**  **(200-400)** | **355.7**  **(200-400)** |

| Inflammatory cytokine | *H. pylori* status | **Tissue isolation site** | **Nonulcer dyspepsia** **(NUD)** | | | | | | | | | | | |
| --- | --- | --- | --- | --- | --- | --- | --- | --- | --- | --- | --- | --- | --- | --- |
|  |  |  | Neutrophils | | | Mononuclear cells | | | Atrophy | | | Intestinal metaplasia | | |
|  |  |  | Mild | Moderate | Severe | Mild | Moderate | Severe | Mild | Moderate | Severe | Mild | Moderate | Severe |
| Mean plasma Interleukins- 6  ng/L | *H. pylori* positive | Antrum | **132.5**  **(10-200)** | **146.5**  **(50-200)** | **230.4**  **(100-300)** | **348.7**  **(150-400)** | **432.8**  **(200-500)** | **502.6**  **(300-600)** | **227.3**  **(150-400)** | **317.4**  **(200-500)** | **514.2**  **(300-600)** | **263.7**  **(150-500)** | **417.2**  **(200-600)** | **512.7**  **(300-600)** |
|  |  | Corpus | **144.3**  **(10-200)** | **152.3**  **(50-200)** | **242.6**  **(100-300)** | **359.2**  **(150-400)** | **445.7**  **(200-500)** | **514.4**  **(300-600)** | **244.7**  **(150-400)** | **335.8**  **(200-500)** | **533.5**  **(300-600)** | **241.6**  **(150-500)** | **401.5**  **(200-600)** | **532.8**  **(300-600)** |
|  | *H. pylori* negative | Antrum | **74.6**  **(10-200)** | **121.5**  **(50-200)** | **164.3**  **(100-300)** | **220.9**  **(150-400)** | **355.7**  **(200-500)** | **403.5**  **(300-600)** | **210.7**  **(150-400)** | **329.5**  **(200-500)** | **482.7**  **(300-600)** | **248.5**  **(150-500)** | **380.6**  **(200-600)** | **476.5**  **(300-600)** |
|  |  | Corpus | **89.3**  **(10-200)** | **134.5**  **(50-200)** | **177.8**  **(100-300)** | **242.6**  **(150-400)** | **368.9**  **(200-500)** | **422.8**  **(300-600)** | **226.4**  **(150-400)** | **346.2**  **(200-500)** | **467.3**  **(300-600)** | **252.6**  **(150-500)** | **392.0**  **(200-600)** | **484.8**  **(300-600)** |

| Inflammatory cytokine | *H. pylori* status | **Tissue isolation site** | **Peptic Ulcer Disease (PUD)** | | | | | | | | | | | |
| --- | --- | --- | --- | --- | --- | --- | --- | --- | --- | --- | --- | --- | --- | --- |
|  |  |  | Neutrophils | | | Mononuclear cells | | | Atrophy | | | Intestinal metaplasia | | |
|  |  |  | Mild | Moderate | Severe | Mild | Moderate | Severe | Mild | Moderate | Severe | Mild | Moderate | Severe |
| Mean plasma Interleukins- 6  ng/L | *H. pylori* positive | Antrum | **124.6**  **(10-200)** | **158.6**  **(50-200)** | **247.8**  **(100-300)** | **363.9**  **(150-400)** | **444.7**  **(200-500)** | **514.8**  **(300-600)** | **249.5**  **(150-400)** | **326.7**  **(200-500)** | **524.9**  **(300-600)** | **272.6**  **(150-500)** | **426.8**  **(200-600)** | **529.7**  **(300-600)** |
|  |  | Corpus | **151.6**  **(10-200)** | **163.8**  **(50-200)** | **255.6**  **(100-300)** | **364.7**  **(150-400)** | **453.6**  **(200-500)** | **526.4**  **(300-600)** | **255.9**  **(150-400)** | **346.8**  **(200-500)** | **541.7**  **(300-600)** | **285.5**  **(150-500)** | **433.9**  **(200-600)** | **542.4**  **(300-600)** |
|  | *H. pylori* negative | Antrum | **95.8**  **(10-200)** | **133.7**  **(50-200)** | **178.2**  **(100-300)** | **231.6**  **(150-400)** | **367.1**  **(200-500)** | **426.4**  **(300-600)** | **227.6**  **(150-400)** | **341.5**  **(200-500)** | **493.7**  **(300-600)** | **257.5**  **(150-500)** | **391.6**  **(200-600)** | **489.5**  **(300-600)** |
|  |  | Corpus | **98.9**  **(10-200)** | **146.9**  **(50-200)** | **189.3**  **(100-300)** | **257.6**  **(150-400)** | **382.9**  **(200-500)** | **448.8**  **(300-600)** | **245.4**  **(150-400)** | **362.2**  **(200-500)** | **481.3**  **(300-600)** | **272.6**  **(150-500)** | **410.0**  **(200-600)** | **512.8**  **(300-600)** |

| Inflammatory cytokine | *H. pylori* status | **Tissue isolation site** | **Gastric Cancer (GC)** | | | | | | | | | | | |
| --- | --- | --- | --- | --- | --- | --- | --- | --- | --- | --- | --- | --- | --- | --- |
|  |  |  | Neutrophils | | | Mononuclear cells | | | Atrophy | | | Intestinal metaplasia | | |
|  |  |  | Mild | Moderate | Severe | Mild | Moderate | Severe | Mild | Moderate | Severe | Mild | Moderate | Severe |
| Mean plasma Interleukins- 6  ng/L | *H. pylori* positive | Antrum | **147.5**  **(10-200)** | **166.5**  **(50-200)** | **242.4**  **(100-300)** | **363.7**  **(150-400)** | **447.8**  **(200-500)** | **523.6**  **(300-600)** | **252.3**  **(150-400)** | **335.4**  **(200-500)** | **528.2**  **(300-600)** | **279.7**  **(150-500)** | **432.2**  **(200-600)** | **525.7**  **(300-600)** |
|  |  | Corpus | **165.3**  **(10-200)** | **172.3**  **(50-200)** | **266.6**  **(100-300)** | **387.2**  **(150-400)** | **469.7**  **(200-500)** | **526.4**  **(300-600)** | **265.7**  **(150-400)** | **352.8**  **(200-500)** | **554.5**  **(300-600)** | **267.6**  **(150-500)** | **426.5**  **(200-600)** | **544.8**  **(300-600)** |
|  | *H. pylori* negative | Antrum | **99.6**  **(10-200)** | **148.5**  **(50-200)** | **187.3**  **(100-300)** | **243.9**  **(150-400)** | **372.7**  **(200-500)** | **428.5**  **(300-600)** | **229.7**  **(150-400)** | **368.5**  **(200-500)** | **512.6**  **(300-600)** | **262.5**  **(150-500)** | **394.6**  **(200-600)** | **492.5**  **(300-600)** |
|  |  | Corpus | **106.3**  **(10-200)** | **164.5**  **(50-200)** | **192.8**  **(100-300)** | **266.8**  **(150-400)** | **395.9**  **(200-500)** | **439.8**  **(300-600)** | **242.8**  **(150-400)** | **384.8**  **(200-500)** | **489.0**  **(300-600)** | **286.5**  **(150-500)** | **409.0**  **(200-600)** | **499.4**  **(300-600)** |

| Inflammatory cytokine | *H. pylori* status | **Tissue isolation site** | **Nonulcer dyspepsia** **(NUD)** | | | | | | | | | | | |
| --- | --- | --- | --- | --- | --- | --- | --- | --- | --- | --- | --- | --- | --- | --- |
|  |  |  | Neutrophils | | | Mononuclear cells | | | Atrophy | | | Intestinal metaplasia | | |
|  |  |  | Mild | Moderate | Severe | Mild | Moderate | Severe | Mild | Moderate | Severe | Mild | Moderate | Severe |
| Mean plasma Interleukins- 8  ng/L | *H. pylori* positive | Antrum | **17.3**  **(10-50)** | **60.8**  **(10-100)** | **148.5**  **(40-200)** | **66.0**  **(10-100)** | **73.5**  **(10-150)** | **128.8**  **(10-200)** | **73.5**  **(10-200)** | **159.7**  **(10-200)** | **251.9**  **(50-300)** | **89.4**  **(50-150)** | **185.2**  **(50-300)** | **225.2**  **(100-300)** |
|  |  | Corpus | **23.6**  **(10-50)** | **74.7**  **(10-100)** | **163.5**  **(40-200)** | **82.6**  **(10-100)** | **96.8**  **(10-150)** | **142.5**  **(10-200)** | **94.5**  **(10-200)** | **172.4**  **(10-200)** | **273.7**  **(50-300)** | **97.3**  **(50-150)** | **196.6**  **(50-300)** | **236.5**  **(100-300)** |
|  | *H. pylori* negative | Antrum | **9.3**  **(10-50)** | **66.7**  **(10-100)** | **134.7**  **(40-200)** | **50.3**  **(10-100)** | **79.7**  **(10-150)** | **83.9**  **(10-200)** | **60.5**  **(10-200)** | **133.4**  **(10-200)** | **220.7**  **(50-300)** | **64.3**  **(50-150)** | **154.3**  **(50-300)** | **214.7**  **(100-300)** |
|  |  | Corpus | **18.2**  **(10-50)** | **82.3**  **(10-100)** | **145.6**  **(40-200)** | **64.9**  **(10-100)** | **82.4**  **(10-150)** | **96.5**  **(10-200)** | **74.6**  **(10-200)** | **147.8**  **(10-200)** | **233.9**  **(50-300)** | **79.4**  **(50-150)** | **172.5**  **(50-300)** | **227.2**  **(100-300)** |

| Inflammatory cytokine | *H. pylori* status | **Tissue isolation site** | **Peptic Ulcer Disease (PUD)** | | | | | | | | | | | |
| --- | --- | --- | --- | --- | --- | --- | --- | --- | --- | --- | --- | --- | --- | --- |
|  |  |  | Neutrophils | | | Mononuclear cells | | | Atrophy | | | Intestinal metaplasia | | |
|  |  |  | Mild | Moderate | Severe | Mild | Moderate | Severe | Mild | Moderate | Severe | Mild | Moderate | Severe |
| Mean plasma Interleukins- 8  ng/L | *H. pylori* positive | Antrum | **19.8**  **(10-50)** | **25.4**  **(10-100)** | **55.8**  **(10-100)** | **78.3**  **(10-100)** | **92.5**  **(10-150)** | **142.8**  **(10-200)** | **95.5**  **(10-200)** | **183.7**  **(10-200)** | **273.9**  **(50-300)** | **110.4**  **(50-150)** | **202.2**  **(50-300)** | **243.2**  **(100-300)** |
|  |  | Corpus | **27.3**  **(10-50)** | **32.7**  **(10-100)** | **75.4**  **(10-100)** | **96.2**  **(10-100)** | **121.3**  **(10-150)** | **167.5**  **(10-200)** | **123.5**  **(10-200)** | **195.4**  **(10-200)** | **295.7**  **(50-300)** | **129.3**  **(50-150)** | **227.6**  **(50-300)** | **248.5**  **(100-300)** |
|  | *H. pylori* negative | Antrum | **12.7**  **(10-50)** | **19.9**  **(10-100)** | **46.7**  **(10-100)** | **66.6**  **(10-100)** | **65.6**  **(10-150)** | **102.2**  **(10-200)** | **88.5**  **(10-200)** | **156.4**  **(10-200)** | **244.7**  **(50-300)** | **87.3**  **(50-150)** | **175.3**  **(50-300)** | **236.7**  **(100-300)** |
|  |  | Corpus | **22.6**  **(10-50)** | **28.4**  **(10-100)** | **58.3**  **(10-100)** | **79.2**  **(10-100)** | **73.4**  **(10-150)** | **114.7**  **(10-200)** | **96.6**  **(10-200)** | **166.8**  **(10-200)** | **258.9**  **(50-300)** | **101.4**  **(50-150)** | **198.5**  **(50-300)** | **249.2**  **(100-300)** |

| Inflammatory cytokine | *H. pylori* status | **Tissue isolation site** | **Gastric Cancer (GC)** | | | | | | | | | | | |
| --- | --- | --- | --- | --- | --- | --- | --- | --- | --- | --- | --- | --- | --- | --- |
|  |  |  | Neutrophils | | | Mononuclear cells | | | Atrophy | | | Intestinal metaplasia | | |
|  |  |  | Mild | Moderate | Severe | Mild | Moderate | Severe | Mild | Moderate | Severe | Mild | Moderate | Severe |
| Mean plasma Interleukins- 8  ng/L | *H. pylori* positive | Antrum | **32.5**  **(10-50)** | **47.4**  **(10-100)** | **76.8**  **(10-100)** | **102.3**  **(10-100)** | **121.5**  **(10-150)** | **166.8**  **(10-200)** | **116.5**  **(10-200)** | **205.7**  **(10-200)** | **295.9**  **(50-300)** | **133.4**  **(50-150)** | **224.2**  **(50-300)** | **267.2**  **(100-300)** |
|  |  | Corpus | **49.3**  **(10-50)** | **54.7**  **(10-100)** | **97.4**  **(10-100)** | **122.2**  **(10-100)** | **143.3**  **(10-150)** | **185.5**  **(10-200)** | **145.5**  **(10-200)** | **214.4**  **(10-200)** | **314.7**  **(50-300)** | **152.3**  **(50-150)** | **246.6**  **(50-300)** | **272.5**  **(100-300)** |
|  | *H. pylori* negative | Antrum | **33.7**  **(10-50)** | **42.9**  **(10-100)** | **68.7**  **(10-100)** | **89.6**  **(10-100)** | **88.6**  **(10-150)** | **134.2**  **(10-200)** | **104.5**  **(10-200)** | **174.4**  **(10-200)** | **268.7**  **(50-300)** | **108.3**  **(50-150)** | **199.3**  **(50-300)** | **261.7**  **(100-300)** |
|  |  | Corpus | **45.8**  **(10-50)** | **53.4**  **(10-100)** | **92.3**  **(10-100)** | **103.7**  **(10-100)** | **98.4**  **(10-150)** | **136.6**  **(10-200)** | **117.6**  **(10-200)** | **188.8**  **(10-200)** | **280.3**  **(50-300)** | **122.2**  **(50-150)** | **219.5**  **(50-300)** | **274.2**  **(100-300)** |

| Inflammatory cytokine | *H. pylori* status | **Tissue isolation site** | **Nonulcer dyspepsia** **(NUD)** | | | | | | | | | | | |
| --- | --- | --- | --- | --- | --- | --- | --- | --- | --- | --- | --- | --- | --- | --- |
|  |  |  | Neutrophils | | | Mononuclear cells | | | Atrophy | | | Intestinal metaplasia | | |
|  |  |  | Mild | Moderate | Severe | Mild | Moderate | Severe | Mild | Moderate | Severe | Mild | Moderate | Severe |
| Mean plasma Interleukins- 10  pg/ml | *H. pylori* positive | Antrum | **34.5**  **(10-100)** | **44.6**  **(10-100)** | **167.2**  **(20-300)** | **237.6**  **(100-300)** | **302.2**  **(100-400)** | **439.8**  **(200-500)** | **410.5**  **(300-500)** | **555.9**  **(300-600)** | **621.8**  **(400-700)** | **314.7**  **(200-500)** | **540.3**  **(300-700)** | **634.7**  **(300-700)** |
|  |  | Corpus | **56.3**  **(10-100)** | **65.6**  **(10-100)** | **189.7**  **(20-300)** | **259.4**  **(100-300)** | **325.6**  **(100-400)** | **462.6**  **(200-500)** | **434.2**  **(300-500)** | **573.5**  **(300-600)** | **644.3**  **(400-700)** | **347.2**  **(200-500)** | **563.1**  **(300-700)** | **657.8**  **(300-700)** |
|  | *H. pylori* negative | Antrum | **27.4**  **(10-100)** | **31.8**  **(10-100)** | **151.3**  **(20-300)** | **216.9**  **(100-300)** | **288.7**  **(100-400)** | **317.7**  **(200-500)** | **374.2**  **(300-500)** | **417.3**  **(300-600)** | **557.9**  **(400-700)** | **295.3**  **(200-500)** | **554.3**  **(300-700)** | **597.2**  **(300-700)** |
|  |  | Corpus | **49.2**  **(10-100)** | **56.3**  **(10-100)** | **174.5**  **(20-300)** | **237.5**  **(100-300)** | **309.6**  **(100-400)** | **339.3**  **(200-500)** | **392.2**  **(300-500)** | **440.7**  **(300-600)** | **579.4**  **(400-700)** | **314.2**  **(200-500)** | **576.2**  **(300-700)** | **619.5**  **(300-700)** |

| Inflammatory cytokine | *H. pylori* status | **Tissue isolation site** | **Peptic Ulcer Disease (PUD)** | | | | | | | | | | | |
| --- | --- | --- | --- | --- | --- | --- | --- | --- | --- | --- | --- | --- | --- | --- |
|  |  |  | Neutrophils | | | Mononuclear cells | | | Atrophy | | | Intestinal metaplasia | | |
|  |  |  | Mild | Moderate | Severe | Mild | Moderate | Severe | Mild | Moderate | Severe | Mild | Moderate | Severe |
| Mean plasma Interleukins- 10  pg/ml | *H. pylori* positive | Antrum | **42.7**  **(10-100)** | **48.6**  **(10-100)** | **173.7**  **(20-300)** | **244.7**  **(100-300)** | **312.1**  **(100-400)** | **462.8**  **(200-500)** | **434.5**  **(300-500)** | **576.2**  **(300-600)** | **646.7**  **(400-700)** | **338.5**  **(200-500)** | **565.3**  **(300-700)** | **656.2**  **(300-700)** |
|  |  | Corpus | **63.4**  **(10-100)** | **71.5**  **(10-100)** | **196.5**  **(20-300)** | **267.4**  **(100-300)** | **334.6**  **(100-400)** | **480.6**  **(200-500)** | **456.2**  **(300-500)** | **597.4**  **(300-600)** | **667.3**  **(400-700)** | **372.2**  **(200-500)** | **587.1**  **(300-700)** | **679.8**  **(300-700)** |
|  | *H. pylori* negative | Antrum | **34.6**  **(10-100)** | **38.4**  **(10-100)** | **162.5**  **(20-300)** | **224.9**  **(100-300)** | **303.7**  **(100-400)** | **338.7**  **(200-500)** | **399.2**  **(300-500)** | **439.7**  **(300-600)** | **579.9**  **(400-700)** | **312.3**  **(200-500)** | **576.3**  **(300-700)** | **616.7**  **(300-700)** |
|  |  | Corpus | **53.2**  **(10-100)** | **62.8**  **(10-100)** | **181.8**  **(20-300)** | **246.7**  **(100-300)** | **332.6**  **(100-400)** | **362.3**  **(200-500)** | **414.5**  **(300-500)** | **465.3**  **(300-600)** | **603.6**  **(400-700)** | **337.4**  **(200-500)** | **598.6**  **(300-700)** | **642.3**  **(300-700)** |

| Inflammatory cytokine | *H. pylori* status | **Tissue isolation site** | **Gastric Cancer (GC)** | | | | | | | | | | | |
| --- | --- | --- | --- | --- | --- | --- | --- | --- | --- | --- | --- | --- | --- | --- |
|  |  |  | Neutrophils | | | Mononuclear cells | | | Atrophy | | | Intestinal metaplasia | | |
|  |  |  | Mild | Moderate | Severe | Mild | Moderate | Severe | Mild | Moderate | Severe | Mild | Moderate | Severe |
| Mean plasma Interleukins- 10  pg/ml | *H. pylori* positive | Antrum | **47.8**  **(10-100)** | **52.2**  **(10-100)** | **177.2**  **(20-300)** | **256.3**  **(100-300)** | **326.8**  **(100-400)** | **459.8**  **(200-500)** | **452.9**  **(300-500)** | **597.9**  **(300-600)** | **667.9**  **(400-700)** | **366.7**  **(200-500)** | **587.3**  **(300-700)** | **674.7**  **(300-700)** |
|  |  | Corpus | **64.9**  **(10-100)** | **72.1**  **(10-100)** | **202.2**  **(20-300)** | **278.1**  **(100-300)** | **343.9**  **(100-400)** | **488.6**  **(200-500)** | **473.4**  **(300-500)** | **573.5**  **(300-600)** | **685.3**  **(400-700)** | **396.2**  **(200-500)** | **617.1**  **(300-700)** | **690.8**  **(300-700)** |
|  | *H. pylori* negative | Antrum | **36.8**  **(10-100)** | **42.7**  **(10-100)** | **167.3**  **(20-300)** | **223.6**  **(100-300)** | **309.6**  **(100-400)** | **339.9**  **(200-500)** | **412.1**  **(300-500)** | **446.3**  **(300-600)** | **598.9**  **(400-700)** | **334.3**  **(200-500)** | **598.3**  **(300-700)** | **639.2**  **(300-700)** |
|  |  | Corpus | **58.2**  **(10-100)** | **64.8**  **(10-100)** | **184.5**  **(20-300)** | **244.9**  **(100-300)** | **334.2**  **(100-400)** | **364.7**  **(200-500)** | **437.9**  **(300-500)** | **488.7**  **(300-600)** | **625.4**  **(400-700)** | **362.2**  **(200-500)** | **621.2**  **(300-700)** | **665.5**  **(300-700)** |
